# Does the fit of personal protective equipment affect functional performance? A systematic review across occupational domains

**DOI:** 10.1371/journal.pone.0278174

**Published:** 2022-11-30

**Authors:** Brooke R. Brisbine, Ceridwen R. Radcliffe, Monica L. H. Jones, Leia Stirling, Celeste E. Coltman

**Affiliations:** 1 University of Canberra Research Institute for Sport and Exercise, Faculty of Health, University of Canberra, Canberra, ACT, Australia; 2 University of Michigan Transportation Research Institute, University of Michigan, Ann Arbor, MI, United States of America; 3 Industrial and Operations Engineering Department, Robotics Institute, University of Michigan, Ann Arbor, MI, United States of America; eCampus University, ITALY

## Abstract

**Objective:**

To explore the effect of personal protective equipment (PPE) fit on functional performance across a range of occupational domains.

**Background:**

PPE introduces an ergonomic, human systems integration, and mass burden to the wearer, and these factors are thought to be amplified if PPE is ill-fitting. However, few studies have considered the role of fit (static, dynamic, and cognitive) when evaluating PPE-related performance detriments in occupational settings.

**Method:**

A systematic literature review was conducted to identify relevant studies, which were then critically appraised based on methodological quality and collated to compare key findings and present evidence-based recommendations for future research directions across a range of occupational domains.

**Results:**

16 published studies met the inclusion criteria, 88% of which found that the fit of PPE had a statistically significant effect on occupational performance. Poorly sized PPE resulted in slower or increased reaction time; decreased range of motion or mobility; decreased endurance or tolerance; decreased pulmonary function; and altered muscle activation. Limited research met the inclusion criteria and those that did had risks of bias in methodology quality.

**Conclusion:**

Future research evaluating the effect of PPE on performance in occupational settings should aim to recruit a more representative population; consider sex as a covariate; quantify and evaluate PPE fit and performance when integrated with all relevant equipment items; include outcome measures related to all three categories of fit (static, dynamic, cognitive); and assess performance of operationally relevant tasks.

## 1. Introduction

Personal Protective Equipment (PPE) describes any item or article worn to minimise risk to the wearer’s health and safety from work-related physical dangers, which might include allergens, ballistic threats, chemicals, electricity, heat, impact, radiological exposure, or sharps [1]. PPE is therefore commonly used in occupational settings that involve exposure to these dangers, such as the military, protective services (e.g. police, firefighting, security), skilled trades (e.g. electrical, plumbing, carpentry), healthcare, and aerospace/aviation. Depending on the particular risks, PPE might include anything from gloves worn by industrial workers in an assembly line (designed to protect the hands from injury and minimise discomfort, particularly when working with hand tools) [2–4], to body armour for military populations (which functions to protect the wearer’s essential organs from ballistic, fragmentation, and stab threats) [5, 6], or space suits (which protect astronauts from the extreme temperatures in space, radiation, and space dust, as well as provide oxygen for astronauts to breathe) [7].

Although protective equipment is the lowest level on the hierarchy of controls for occupational hazards [8], PPE is essential in many occupational domains and required to comply with safety and protection standards [9]. However, PPE often introduces an ergonomic, human systems integration, and mass burden to the wearer [10]. Use of body armour systems and firefighter turnout gear, for example, have been quantitatively shown to negatively impact ROM and dynamic task performance [11–15]. Working in hot and humid conditions while wearing PPE has also been shown to place additional physiological stress on the body that impacts cognition and comfort, ultimately leading to fatigue, decreased performance, and injury [9, 16, 17]. Strategies are therefore needed to minimise detriments associated with requisite PPE use.

Fit is thought to be key factor affecting functional and operational performance detriments associated with PPE use [18]. Correctly sized PPE has been consistently shown to minimise ROM loss [14, 19], interference [20], physiological stress, and fatigue [21] associated with the use of protective equipment. Conversely, improperly sized PPE increases the likelihood of overexertion, fatigue, discomfort, and injury [22, 23]. For example, research has demonstrated that undersized body armour may compromise protection to essential organs [6], while oversized body armour, although likely to increase protection, has been shown to negatively impact mobility and operational task performance [5, 14, 20]. However, optimal sizing and associated fit is often difficult to achieve. In the case of female soldiers, for instance, several recent publications have reported that the existing unisex sizing range does not accommodate the variety of female breast and torso shapes among the population and therefore that women in this field are more likely to be affected by fit-related PPE detriments [13, 20, 23]. Studies of firefighting uniforms have reached similar conclusions, reporting that oversized pants and jackets have a significant negative effect on range of motion and performance compared to pants and jackets that are correctly sized [11, 24]. Interestingly, female participants in one particular firefighting study reported that they intentionally ordered pants larger than their recommended uniform size to accommodate their proportionally larger hips, which had the unfortunate consequence of limiting their range of motion and impairing task performance [12]. These examples of PPE sizing affecting occupational task performance, particularly for women, are not isolated and have been broadly reported across domains from aerospace to manufacturing to healthcare [25–32]. Sizing and associated fit should therefore be an essential consideration when evaluating the effect of PPE on performance.

The concept of “fit” represents an optimized status between the wearer and their immediate environment [33]. Beyond this, however, fit has been poorly defined in the literature in relation to protective equipment. Although specific to functional wearable equipment such as exoskeletons, a recent review by Stirling et al. proposes the most complete definition of fit and its various classifications [18]. Specifically, these include *static fit*, or the alignment between the wearer’s anthropometry (three-dimensional size and shape) and the PPE system; *dynamic fit*, or how the wearer and the system interact during functional ROM and task performance; and *cognitive fit*, or how the wearer’s cognitive and decision-making capabilities are impacted by wearing the system [18]. Each of these fit characteristics are influenced by the others in a complex interaction; however, the development of PPE sizing has focused, perhaps disproportionately, on *static fit* and how a limited number of standard anthropometric characteristics of soldiers, police officers, firefighters, or healthcare workers align with dimensions of the PPE system [5, 14, 19, 20, 25, 27–30, 32, 34].

Typically, “fit” has been linked to the existing sizing based on a two-step process, whereby (1) anthropometric dimensions (e.g. torso length) are used to determine a recommended size and (2) subsequent subjective assessments (e.g. participant comfort) result in shifting up or down in sizing to achieve the best fit based on the range of sizes available. This method informs which size was selected by the process, as well as provides insight into subjective issues that resulted in deviations from the recommended size, but ultimately fails to identify specific dimensions associated with ill fit due to the large number of confounding variables. Indeed, as multiple dimensions associated with the PPE are changed simultaneously based on the original sizing concept, it is difficult to discern the source of ill fit and therefore recommendations for future PPE design are limited [35, 36]. While one-dimensional anthropometric measures coupled with user perceptions can support initial sizing, there is opportunity to adapt the nominal two-step process by expanding initial sizing and PPE evaluation to include a comprehensive assessment of fit inclusive of three-dimensional static fit, human movement in the system (dynamic fit), and the potential mental impact of system use (cognitive fit). Through assessment of static, dynamic, and cognitive fit in combination, organisations can better understand and alleviate functional performance detriments related to PPE system use within their respective occupational settings.

Despite the breadth of previous research that has quantified the impact of PPE on human performance (i.e. compared to a baseline condition without any PPE), relatively few published studies have considered the effect of PPE fit in performance evaluations (i.e. comparing fit conditions) and there remain many gaps in the current knowledge [11, 24, 37–42]. Therefore, the aims of this systematic review were to (i) characterise methods of investigating the effect of PPE fit on performance; (ii) synthesise previous findings of PPE fit on a range of performance measures across occupational domains; and (iii) identify research problems regarding PPE fit and occupational performance to recommend future research directions.

## 2. Materials and methods

### 2.1 Literature search

The following electronic scientific databases were searched from study inception to May 2022: MEDLINE, SCOPUS, PUBMED, CINAHL, Science Direct, Web of Science. Search terms were piloted and reviewed across each database; the specific advanced search terms with relevant Boolean operators are shown in Table 1. As government-funded military and aerospace research is commonly presented at conferences, a hand search of the International Conference on Environmental Systems congress proceeding databases was also conducted to identify any additional literature.

**Table 1:** The search strategy terms to identify studies relevant to the present review.

Terms						
PPE		Occupational Domain		Performance measure		Fit criteria
“Personal protective equipment” OR “protective equipment” OR PPE OR equipment OR “protective clothing” OR “body borne” OR body-borne OR suits OR gloves OR “tactical armour” OR “space suit” OR spacesuit OR exoskeleton OR “individual protective equipment” OR “tactical vest” OR exosuit OR exosystem OR gear OR “safety vest” OR “safety equipment” OR turnout OR kit OR “fall vest” OR harness OR armor OR armour OR “equipment configurations” OR boots OR “body armor” OR “body armour”)	AND	(Military OR “emergency services” OR security OR defence OR defense OR police OR “law enforcement” OR medical OR firefighter OR officers OR aerospace OR space OR “emergency response” OR soldiers OR paramedics OR “fire service” OR astronaut OR cosmonaut)	AND	(“physical performance” OR performance OR task OR movement OR “range of motion” OR “physical fitness” OR balance OR “joint angles” OR “operational task performance” OR cognitive OR strength OR “joint displacement” OR “range of movement” OR flexibility OR “task performance” OR ROM OR pressure OR “contact pressure” OR attention OR “situation awareness” OR memory OR “situational awareness”)	AND	(fit OR “static fit” OR “dynamic fit” OR “task specific fit” OR “cognitive fit” OR ease OR fall)

### 2.2 Inclusion and exclusion criteria

**Fig 1:**
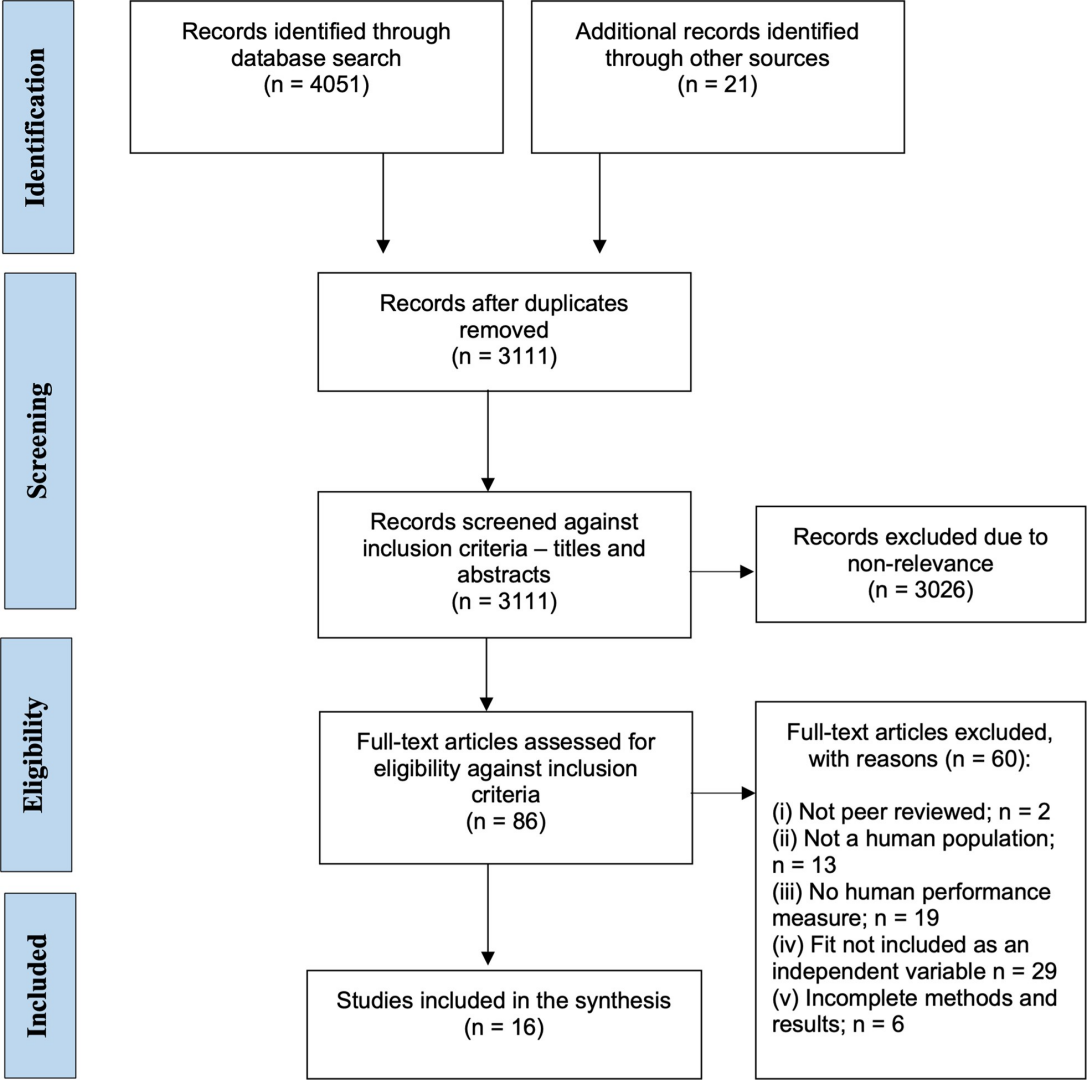
PRISMA flow diagram [43] of the study selection, including literature search and reasons for exclusion. The reason for exclusion of an article was based on a hierarchy; that is, where a paper failed to meet multiple inclusion criteria, it was excluded based on the first appropriate reason and counted at this point in the exclusions list.

This systematic review was conducted in accordance with the guidelines from the Preferred Reporting Items for Systematic Reviews and Meta-Analysis (PRISMA) [43]. The flow chart shown in Fig 1 illustrates the results of the literature search, screening, and selection process of studies for inclusion in the present review. Specifically, all identified references were imported into Covidence Systematic Review Software (Melbourne, Australia) and duplicates were removed. Studies identified by the initial search (n = 3111) were then assessed based on title and abstract by two authors (CC and CR) separately and independently in Covidence, and any discrepancies between the two reviewers were discussed until consensus was reached. Studies were only eligible for inclusion in this review if they: (i) incorporated at least one measure of fit (static, dynamic, or cognitive) of the PPE item; (ii) investigated the effects of PPE on at least one measure of human performance (including physical or cognitive assessments); (iii) considered a specific occupational domain; (iv) were published in English; and (v) were published after 1970, as relevant legislation mandating the use of PPE was passed in 1974 (Health and Safety Work Act 1974, Australia). Based on a review of the title and abstracts against this inclusion criteria, 86 studies were assessed as full-text by two of the authors (CC and CR) to determine relevance to the present review. At this stage. studies were excluded if they: (i) were not peer reviewed; (ii) did not recruit a human population; (iii) did not include an appropriate human performance measure (note: although this was specified as inclusion criteria for full-text screening, it was not always apparent from the title and abstract); (iv) did not include fit as an independent variable; or (v) did not present complete methods and results. Based on these exclusion criteria, sixteen studies were included in the review.

### 2.3 Data extraction

Data were extracted from the 16 included studies into an Excel database (Microsoft, USA) by two authors (BB and CR) and collated to provide a systematic overview of main findings, establish the strength of available evidence, and identify gaps in current knowledge. Key data extracted included participant characteristics (e.g. sample size, sex, age, occupational domain, country), equipment characteristics (e.g. type of equipment used, user experience, sizing, designs), study characteristics (e.g. design, setting, test conditions, fit assessment, performance measures), and a summary of findings (e.g. effect of PPE fit on performance).

### 2.4 Risk of bias analysis

Eligible studies identified through the literature search and screening processes were critically appraised to assess methodological quality using the Mixed Methods Appraisal Tool [44] The Mixed Methods Appraisal Tool includes specific criteria for qualitative, quantitative and mixed methods studies, focusing on methodological quality (Table 3). Each study included in the quality appraisal was evaluated by two reviewers (CC and CR). Every study was assigned a score (0–2) based on each question within the appraisal tool, with a rating of 2 adopted to indicate a low risk of bias, a rating of 1 indicating an unclear risk of bias, and a rating of 0 indicating a high risk of bias. These ratings were documented and included in the results.

## 3. Results and discussion

A total of 16 studies [5, 12, 14, 19–21, 25–32, 34, 45] met the inclusion and exclusion criteria for incorporation in the present review: 13 were non-randomised studies, one was a quantitative descriptive study, and two were mixed-methods studies (Table 3). Notably, a wide range of papers that assessed the effect of PPE on performance without including fit as an independent variable were excluded from the review [11, 24, 37–42]. Publications originated from four countries: USA (n = 10) [5, 12, 14, 25–29, 34, 45]; UK (n = 4) [19, 21, 30, 31]; Australia (n = 1) [20]; and Korea (n = 1) [32]. A methodological overview (Table 2), quality appraisal (Section 3.5; Table 3), and key findings (Section 3.6; Table 4) of each of the 16 studies included in this review are summarised and discussed below. Corresponding recommendations for future research are addressed in Section 3.7.

**Table 2:** Methodological overview of the 16 studies included in the present review.

Study	Occupational domain	Aim	N	Participants	PPE tested	Size selection	Fit conditions	Testing protocol	Performance measure(s)	Evaluation of fit
Armstrong et al. 2019 [21]	Military	To define the physiological response to wearing body armour with loads of varying masses on the soldier and identify the conditions under which soldiers maybe become susceptible to respiratory muscle fatigue and expiratory flow limitation	24	Male infantry soldiers	United Shields T45 modular tactical vest	Five sizes of body armour available from S–XXL and size was determined by researchers. Backpack size was one size. Body armour and backpack straps were fastened by researcher while participant inhaled to total lung capacity to determine loose fit.	• Regular fit • Loose fit	After a 10min rest, participants walked or ran on a treadmill, the incline and speed of which increased every 10min to simulate cautious patrol (light exercise; low threat patrol (moderate exercise); forced march (heavy exercise); a contact situation (very heavy exercise)	Pulmonary ventilation; heart rate; perceived exertion	Dynamic
Choi et al. 2016 [5]	Military	To investigate how wearing body armour and the fit of that body armour affects marksmanship performance	15	Male active-duty soldiers	U.S. Army standard-issue Improved Outer Tactical Vest (IOTV) Generation III body armour system, and Advanced Combat Helmet	Initial body armour size was determined by a fit expert, via visual inspection	• No body armour • Duty uniform only (baseline) • Initial (best fit) body armour size • One size smaller • One size larger	Participants performed a marksmanship task (single and multiple targets) using a weapon simulator	Mobility, speed, marksmanship accuracy, precision	Dynamic, Cognitive
Choi et al. 2018 [14]	Military	To examine the effect of body armour fit on Warfighter mobility and ROM	40	Male active-duty US Army military personnel	Generation III Improved Outer Tactical Vest (IOTV)	Predicted size was determined based on Chest Circumference, after which a subject matter expert evaluated the coverage of the ballistic plate and soft armour when standing and seated	• Semi-nude • Duty uniform • Initial (predicted) fit size • Between 1–3 other sizing configurations (increased, decreased & longer length)	Participant range of motion was measured for: upper arm/shoulder abduction; upper arm/shoulder forward extension; cervical rotation, ventral-dorsal cervical flexion; cross body extension; thoracic/lumbar spine rotation; thoracic/lumbar spine lateral flexion; trunk flexion–standing; trunk flexion–seated; overhead fingertip reach–standing; forward extended reach–standing; high knee; cross body reach–sitting	Range of motion	Dynamic
Coltman et al. 2020 [20]	Military	To determine the prevalence of ill-fitting body armour and the effects of ill-fitting armour on the functional attributes of musculoskeletal pain and discomfort, integration with other soldier equipment and mobility when female soldiers completed operationally-representative tasks	147	Female Australian regular army soldiers	Tiered Body Armour System (Tier 2 and Tier 3)	Size was not reported. Participants provided feedback on their current-issue system and these data were used to group participants into three fit conditions	• Too small • Good fit • Too large	Participants completed a questionnaire about the fit and function of their body armour	Pain & discomfort questionnaire, perception of movement interference, marksmanship/driving performance that requires coordination, attention & executive functioning	Static, Dynamic, Cognitive
Davis et al. 2020 [19]	Military	To identify sex-specific issues with body armour related to fit and function	150	Female armed forces personnel	OSPREY body armour	Participants specified their current issue body armour size, bra size (converted to chest circumference) and stature, which were compared to the OSPREY sizing chart and used to classified whether the participant was wearing the correct size	• Body armour fit to chest but not stature • Fit to stature but not chest • Fit to both stature and chest	Participants completed a questionnaire about discomfort and functionality of their body armour, as well as their perception of functional movement	Discomfort questionnaire, functional movement task	Static, Dynamic
Drabek et al. 2010 [25]	Healthcare	To evaluate the effect of wearing the wrong sized gloves on tasks involving manual dexterity	20	Male & female medical students, resident anaesthesiologists, and nurses	Standard latex surgical gloves (Triflex Custom; Cardinal Health, McGraw Park, Ill)	Participants self-selected their preferred glove size based on prior clinical experience	• Just right (preferred size) • Too small (full size smaller) • Too large (full size larger) • Bare handed	Participants performed the grooved pegboard test to assess manual dexterity and neuropsychological factors	Dexterity, speed, pegboard test performance	Dynamic, Cognitive
Drabek et al. 2013 [34]	Healthcare	To investigate the effect of non-sterile, ambidextrous vinyl examination gloves on manual dexterity when compared with bare hands, irrespective of their size	20	Male & female medical students, anaesthesia residents, nurses, and hospital support staff	Non-sterile, ambidextrous, vinyl examination gloves	Participants self-selected their preferred glove size based on prior clinical experience	• Preferred glove size ’just right’ • One size smaller ’too small’ • One size larger ’too large’	Participants performed the grooved pegboard test to assess manual dexterity and neuropsychological factors	Dexterity, speed, pegboard test performance	Dynamic, Cognitive
Fineman et al. 2018 [26]	Aerospace	To evaluate the sensitivity of proposed new metrics to detect how changes from nominal static fit (padding at the hip and thigh) cause potential changes in dynamic fit in the lower extremities during a simple gait task	3	General population aligned with astronaut eligibility criteria	MKIII Spacesuit	Participants wore an MKIII spacesuit and volumetric scans were used to determine the level of padding added between the subject hips/ thighs and MKIII hip briefs for the various fit conditions	• No padding • Single layer of padding • Double layer of padding	Participants completed a series of walking tasks on an elevated walkway	Knee range of motion; cadence	Dynamic
Hearon et al. 1998 [27]	Aerospace	To examine gender differences in +Gz endurance to a more operational +Gz profile (SACM) in subjects wearing best-fit antiG suits	14	Male & female members of the of the Brooks AFB Acceleration Research Subject Panel	CSU-13B/P antiG suit (OTO suit) or AL Mod suit, as this was best fit for women	Participants were sized initially by height and weight tables established in the original technical order suits, then with modifications (vertical waistband reduction and/or the "v" dart in the waistband)	• ’Best fit’ suit • Modified suit	Participants performed a +5.0 to +9.0 Gz centrifuge-simulated air combat maneuver to fatigue using the anti-G straining maneuver with anti-G suit inflation	Maximal endurance of +5.0 to +9.0 Gz (time to fatigue)	Dynamic, Cognitive
Lombardo et al. 2020a [29]	Aerospace	To evaluate whether static fit metrics derived from glove dimension and human anthropometry are related to spacesuit glove performance on a general tactility task, and an operationally relevant tactility task	9	University students	Prototype gloves, similar in design to the DCCI Orion Crew Survival System (OCSS) IVA gloves	Prescribed size was determined by direct measures (length and circumference of the hand, index finger and thumb) and perceived size using 2 surveys	• One size below prescribed fit • Prescribed fit size • One size larger than prescribed fit	Participants completed a battery of functional assessment tasks (including a generalized tactility task and an operationally-relevant tactility task) in a glovebox vacuum chamber.	Tactility assessment	Dynamic, Cognitive
Lombardo et al. 2020b [28]	Aerospace	To evaluate the effect of static fit on dexterity, technical flight performance, and mental workload.	9	University students ranging from no flight experience to licensed pilots	Prototype gloves, similar in design to the DCCI Orion Crew Survival System (OCSS) IVA gloves	Prescribed size was determined by direct measures (length and circumference of the hand, index finger and thumb) and perceived size using 2 surveys	• One size below prescribed fit • Prescribed fit size • One size larger than prescribed fit	Participants completed several dexterity tasks (U-Bolt Pegboards, knot-tying and bow-tying), mobility and grip strength tasks, as well as the Draper real-time performance metrics workstation lunar landing simulator to assess flight performance, mental workload and situational awareness	Dexterity, technical error, mental workload; Situational awareness	Dynamic, Cognitive
McCloskey & Esken 1995 [45]	Aerospace	To quantify effects of high G forces (sustained acceleration) on I-NIGHTS component integrity, migration of the intensified image in relation to the human field of view, the effects of neck strength on subjects’ ability to keep a head-mounted target steady, and subjective opinions concerning each system	10	Male & females on the Acceleration Subject Panel	3x I-NIGHTS helmet types: GEC; Honeywell; and Kaiser	Participants wore 3 different brands of helmet and were assessed as either a pass or fail	• Fit-pass • Fit-fail	Participants were exposed to four different high-G profiles on a man-rated centrifuge and neck strength was evaluated for all helmet conditions	Strength assessment	Dynamic
Mylon et al. 2016 [30]	Healthcare	To validate three test methods as tools for glove evaluation	18	Male & female university students	POLYCOHealthcare ambidextrous examination gloves: Finex powder free latex gloves (chlorinated on the outside surface), Finity powder free (PF) vinyl gloves	Participants were allowed to choose the size of glove that fitted them best, with some advice from the researcher (since most had little or no experience of wearing examination gloves)	• Best fit • 1–2 sizes larger than best fit	Participants completed the Purdue Pegboard Test, Crawford Small Parts Dexterity Test and Semmes-Weinstein Monofilaments	Speed; accuracy; dexterity; tactility; pegboard test performance	Dynamic, Cognitive
Park & Langseth-Schmidt 2016 [12]	Firefighting	To identify fit issues of the female firefighters’ uniform and determine specific areas on the uniform that cause fit issues based on a comprehensive anthropometric fit analysis using 3D body scanning technology, survey, and interview methods	18	Male & female career and volunteer firefighters	Firefighting uniform pants	Participants wore their current uniform pants	• Baseline (undergarments) • Station pants with a basic t-shirt • Turnout pants worn over station pants with a basic t-shirt	Participants completed a subjective comfort survey, 3D body scan, and exit interview	Fit (functional ease based on 3D body scan & using an equation); subjective perceptions of fit	Static, Dynamic
Stevenson et al. 2013 [31]	Aerospace	To investigate whether variation in FCAGT garment fit might compromise transmission of intra-garment pressure sufficiently to degrade physiological protection from +Gz and thus aircrew +Gz tolerance	6 / 8	Male experienced centrifuge subjects	FCAGT (Full coverage anti G trouser)	Participants were fitted by a subject matter expert into a fully fitted suit and then partially fitted suits; occasionally, loose fits over the abdomen, thighs, and calves were achieved by adjusting suit fit while wearing shaped pads (2cm thick)	• Fully fitted • Abdomen only fitted • Legs only fitted • Loose fit	Participants sat in a chair with a four-point harness and performed a series of spirometry tests while wearing Full-Coverage Anti-G Trousers	Pulmonary function; heart rate	Dynamic
Yoo et al. 2011 [32]	Manufacturing & assembly	To assess the effects of wearing the wrong size glove on EMG activity in the shoulder and forearm muscles when performing simulated model-assembly operations	16	Asymptomatic seated workers	Nitrile Exam gloves are 9-inch length, nonsterile, powder free and ambidextrous gloves	Well-fitting gloves were determined by direct measures of the width of thumb, index and middle finger at the halfway point on the distal phalange and the length of thumb, index and middle finger	• Bare hands • Well-fitting gloves • One size smaller gloves • One size larger gloves	Participants completed the Valpar Component Work Sample No.204 task in each glove condition with an EMG attached to the skin	Muscle activation of upper trapezius, serratus anterior, flexor digitorum superficialis, extensor digitorum; operational task performance	Dynamic, Cognitive

**Table 3:** Quality assessment of the included 16 studies, based on the Mixed Methods Appraisal Tool criteria (MMAT) [44]. A score of 0 indicates a high risk of bias (dark grey), a score of 1 indicates an unclear risk of bias (grey) and a score of 2 indicates a low risk of bias (light grey); a description of the 7 checklist items is presented for each type of study in the figure legend. NB: Studies labelled in italics are Mixed-Methods (Davis et al. 2020; Park & Langseth-Schmidt et al. 2016); as per the MMAT [44], these studies were assessed on both Qualitative and Quantitative /Non-Randomised study criteria below.

Study Type	Checklist item						
Qualitative Studies*	1 Clear research question	2 Data address question	3 Appropriate design	4 Data collection	5 Findings from data	6 Result interpretation	7 Coherence
*Davis et al. 2020 [19]*	2	2	2	2	2	2	2
*Park & Langseth-Schmidt 2016 [12]*	2	2	2	0	2	2	2
**Non-Randomised Studies** ^**†**^	1 Clear research question	2 Data address question	3 Representative participants	4 Appropriate measurements	5 Outcome data	6 Confounders accounted for	7 Intervention administered
Armstrong et al. 2019 [21]	2	2	2	2	2	2	2
Choi et al. 2016 [5]	2	2	2	2	2	1	2
Choi et al. 2018 [14]	2	2	2	2	2	2	2
Drabek et al. 2010 [25]	2	2	2	2	2	2	2
Drabek et al. 2013 [34]	2	2	2	2	2	1	2
Fineman et al. 2018 [26]	2	2	0	2	2	2	2
Hearon et al. 1998 [27]	2	2	1	2	2	2	2
McCloskey & Esken 1995 [45]	2	2	1	2	2	2	2
Lombardo et al. 2020a [29]	2	2	0	2	2	2	2
Lombardo et al. 2020b [28]	2	2	0	2	2	2	2
Mylon et al. 2016 [30]	2	2	0	2	0	1	2
Stevenson et al. 2013 [31]	2	2	1	2	2	2	2
Yoo et al. 2011 [32]	2	2	2	2	2	2	2
**Quantitative Descriptive Studies** ^**‡**^	1 Clear research question	2 Data address question	3 Sampling strategy	4 Representative sample	5 Appropriate measures	6 Low risk of nonresponse	7 Appropriate stats
Coltman et al. 2020 [20]	2	2	2	2	2	2	2
*Davis et al. 2020 [19]*	-	-	2	2	1	2	2
*Park & Langseth-Schmidt 2016 [12]*	-	-	2	0	2	2	0
**Mixed-Methods Studies** ^**§**^	1 Clear research question	2 Data address question	3 Appropriate design	4 Methods integrated	5 Adequately interpreted	6 Inconsistencies addressed	7 Quality criteria
*Davis et al. 2020 [19]*	-	-	2	2	2	2	2
*Park & Langseth-Schmidt 2016 [12]*	-	-	2	2	0	1	0

* 1 –Are there clear research questions? 2 –Do the collected data allow to address the research questions? 3—Is the qualitative approach appropriate to answer the research question? 4—Are the qualitative data collection methods adequate to address the research question? 5—Are the findings adequately derived from the data? 6—Is the interpretation of results sufficiently substantiated by data? 7—Is there coherence between qualitative data sources, collection, analysis and interpretation?

^**†**^ 1 –Are there clear research questions? 2 –Do the collected data allow to address the research questions? 3—Are the participants representative of the target population? 4—Are measurements appropriate regarding both the outcome and intervention (or exposure)? 5—Are there complete outcome data? 6—Are the confounders accounted for in the design and analysis? 7—During the study period, is the intervention administered (or exposure occurred) as intended?

^**‡**^ 1 –Are there clear research questions? 2 –Do the collected data allow to address the research questions? 3—Is the sampling strategy relevant to address the research question? 4—Is the sample representative of the target population? 5—Are the measurements appropriate? 6—Is the risk of nonresponse bias low? 7—Is the statistical analysis appropriate to answer the research question?

^**§**^ 1 –Are there clear research questions? 2 –Do the collected data allow to address the research questions? 3—Is there an adequate rationale for using a mixed methods design to address the research question? 4—Are the different components of the study effectively integrated to answer the research question? 5—Are the outputs of the integration of qualitative and quantitative components adequately interpreted? 6—Are divergences and inconsistencies between quantitative and qualitative results adequately addressed? 7—Do the different components of the study adhere to the quality criteria of each tradition of the methods involved?

**Table 4:** Key findings from the 16 studies included in the present review regarding the effect of PPE fit on performance of (A) body armour; (B) gloves; (C) helmets; (D) spacesuits; and (E) uniforms.

Study	Occupational domain	Sig. effect of PPE on performance	Sig. effect of PPE fit on performance	Effect of PPE fit on performance
**(A) Body Armour**				
Armstrong et al. 2019 [21]	Military	Yes	Yes	Maximal voluntary ventilation was reduced by 11% in the loose armour configuration compared to battle-fit, but no other differences in spirometry, cardiovascular parameters, mouth pressures, or operating lung volumes were observed.
Choi et al. 2016 [5]	Military	Yes	Yes	Accuracy and precision were not significantly affected by body armour fit; however, speed was degraded in the initial fit body armour and the increased size configurations relative to the baseline and decreased size configurations.
Choi et al. 2018 [14]	Military	Yes	Yes	All measures of range of motion were decreased in the initial fit compared to no body armour. Smaller body armour and initial fit were very similar but larger body armour decreased ROM.
Coltman et al. 2020 [20]	Military	Did not assess	Yes	Body armour that was too small or too large was associated with increased severity of musculoskeletal pain, amplified integration issues with other soldier equipment, and increased interference when performing a range of operationally representative tasks.
Davis et al. 2020 [19]	Military	Did not assess	Yes	Poor fit at the shoulder restricts range of motion of the arms, while poor chest fit restricts breathing and the ability to reach hips/pouches on armour/trouser pockets.
**Study**	**Occupational domain**	**Sig. effect of PPE on performance**	**Sig. effect of PPE fit on performance**	**Effect of PPE fit on performance**
**(B) Gloves**				
Drabek et al. 2010 [25]	Healthcare	No	Yes	Wearing too small gloves or too large gloves increased the time it took to complete the pegboard test, compared to gloves that fit just right (preferred size) or bare-handed performance of the task.
Drabek et al. 2013 [34]	Healthcare	No	No	Time to remove pegs was decreased when wearing the preferred glove size compared to completing the task with bare hands.
Lombardo et al. 2020a [29]	Aerospace	Yes	Yes	Direct measures of static fit derived from hand length and glove length had a significant relationship to performance on the switchboard tactility task. Additionally, it was found that in the unpressurized case, subjects performed significantly better on the switchboard task when wearing the larger gloves with more easement. There was no difference in pressurized performance across the sizes examined for either tactility task.
Lombardo et al. 2020b [28]	Aerospace	Yes	Yes	Prescribed glove size resulted in significantly slower response time than the small glove.
Mylon et al. 2016 [30]	Healthcare	Yes	Yes	Wearing no gloves resulted in the best performance of the Purdue Pegboard Test, while best fit in either type of glove came in second, followed by double layer of best fit gloves, and lastly larger gloves, which were worst for performance.
Yoo et al. 2011 [32]	Manufacturing & assembly	Yes	Yes	Wearing the wrong size glove led to decreased forearm muscle activation, which resulted in increased shoulder movements. Bare-hands or well-fitting gloves caused effective forearm muscle activation, which decreased inefficient shoulder movements.shoulder movements. Bare-hands or well-fitting gloves caused effective forearm muscle activation, which decreased inefficient shoulder movements.
**Study**	**Occupational domain**	**Sig. effect of PPE on performance**	**Sig. effect of PPE fit on performance**	**Effect of PPE fit on performance**
**(C) Helmet**				
McCloskey & Esken 1995 [45]	Aerospace	Did not assess	No	Helmet fit had no significant effect on performance measures of neck strength.
**Study**	**Occupational domain**	**Sig. effect of PPE on performance**	**Sig. effect of PPE fit on performance**	**Effect of PPE fit on performance**
**(D) Spacesuit**				
Fineman et al. 2018 [26]	Aerospace	Yes	Yes	Padding added to alter suit fit at the hip/thighs had mixed effects on gait performance and dynamic fit measures, suggesting that dynamic fit between subjects may be more reliant on alternate aspects of fit, such as suit component sizes and designs.
Hearon et al. 1998 [27]	Aerospace	Did not assess	Yes	Females were able to endure the centrifuge-simulated air combat maneuver for nearly double the time in the modified suit than they could in the ‘best fit’ suit based on the sizing chart.
**Study**	**Occupational domain**	**Sig. effect of PPE on performance**	**Sig. effect of PPE fit on performance**	**Effect of PPE fit on performance**
**(E) Uniform**				
Park & Langseth-Schmidt 2016 [12]	Firefighting	Did not assess	Yes	Female firefighters experienced poorer fit and a higher level of discomfort with their uniform pants than male firefighters, which was associated with reduced functional ease in range of motion.
Stevenson et al. 2013 [31]	Aerospace	Did not assess	Yes	Relaxed 1Gz Tolerance was significantly affected by Full-Coverage Anti-G Trouser fit. Loose fit results in increased garment volume and a decrease in the pressure-volume ratio, and is associated with a significantly greater lung volume. A loose abdominal fit, with or without a loose fit over the lower limbs, resulted in a marked decrease in tolerance to 1Gz acceleration and an increase in perceived effort under sustained high 1Gz acceleration.

### 3.1 Occupational domain

Included studies represented a range of occupational domains, including aerospace (n = 6) [26–29, 31, 45]; military (n = 5) [5, 14, 19–21]; healthcare (n = 3) [25, 30, 34]; firefighting (n = 1) [12]; and manufacturing and assembly (n = 1) [32]. Despite the frequent use of PPE in biological [46], chemical [47], or anatomical laboratories [48], no publications from these domains met the inclusion criteria for the present review, nor did any studies related to the construction [49], mining [50], or agricultural [51] industries.

### 3.2 Participants

#### 3.2.1 Sample size

A total of 527 participants were involved across the 16 studies, with an average of 33 participants per study. The sample size ranged broadly between studies from 3 participants [26] to 150 participants [19]), and more than 81% (n = 13) [5, 12, 21, 25–32, 34, 45] recruited fewer than 30 participants. Small sample sizes limit the generalisability of the current literature within the specific occupational domain, as they are unlikely to have adequate statistical power to account for human variability (e.g., anthropometry, posture, experience, and self-selected approach to task performance). Therefore, it is recommended that future research recruit larger, more representative samples to ensure that results accurately characterise the user population.

#### 3.2.2 Sample population

A majority of studies within the present review recruited participants that were specific to the occupational domain (n = 12; 75%) as opposed to general population (university students [28–30] or adults who met general astronaut eligibility criteria [26]; n = 4; 25%). Considering that workers within a given occupational domain are likely to have characteristics and skills unique to their profession, as well as experience using the equipment item, recruiting participants from the general population may confound results and limit generalisation, for example, to actual manufacturing and assembly workers [32], to healthcare workers [30], or to astronauts [26]. A further limitation of current studies is that few were actively sampled to represent the user population; that is, sampling a range of body size and shape dimensions representative of the user population and that would interact with and vary overall PPE fit, such as BMI, breast size, or hand dimensions. As such, many study samples fail to represent the diversity of the user population, thus limiting the generalisability of results. To ensure ecological validity of study results, it is therefore recommended that representative participants are recruited from the intended end-user population [52–54], with consideration for sampling across the relevant anthropometric distributions.

#### 3.2.3 Participant age

Of the studies that reported a mean age of all participants (n = 10), the average age was 27.7 years; an additional three studies reported separate mean ages for male and female participants [12, 27, 31], two studies only reported the age range [30, 45], and one neglected to report any data on participant age.[19] Ensuring that participant age is representative of the user population is important given the association between age and human performance [55–57]. For example, evaluating the dexterity of university students (aged 21–30 years) while wearing gloves will have limited application for healthcare workers above that age range [30]. It is recommended that future research recruit participants from a broad age-range, reflective of the end user population to ensure generalisability of the results to the entire user population.

#### 3.2.4 Sex differences

Half of the included studies recruited a mix of male and female participants (n = 8; 50%)[12, 25, 27–30, 34, 45], with fewer studies recruiting male-only participants (n = 5; 31%) [5, 14, 21, 26, 31], and the fewest number of studies recruiting female-only participants (n = 2; 13%) [19, 20]. One study did not report the sex of participants, specifying only that participants were “sixteen asymptomatic seated workers with normal hands and no deformities, skin diseases, or latex allergies” [32]. Given that previous research has demonstrated a marked difference in the performance detriments that males and females experience when using PPE [58, 59], as well as observing varied anthropometric characteristics [60] and physiological responses between the sexes [61, 62], the generalisability of performance results within the profession are likely to be limited if studies do not consider sex as a covariate or if sex is not reported at all.

Furthermore, existing research indicates that females across a range of occupations experience increased fit issues with PPE compared to their male counterparts [12, 20], which often translates to amplified performance effects. Despite this finding, females remain underrepresented in literature pertaining to protective equipment. For example, male firefighters may experience no significant performance detriments when wearing properly sized turnout gear, while the functional ease and mobility of female firefighters are greatly compromised [12]; yet, access to female-specific firefighting PPE is low (42% of 840 female firefighters surveyed in the UK and Ireland, North America, Australasia and mainland Europe) [63]. Similar results in terms of sex-specific performance detriments have been reported across a range of occupations [12, 27, 58, 59]. Four studies (of the eight that recruited mixed sex participants) included only one [29] or two [28, 30, 45] female participants, which makes a valid statistical comparison between sexes impossible. Indeed, only two studies compared either performance effects or fit-related performance effects between men and women [12, 27], but despite substantial anecdotal evidence, the extent of sex or gender differences in many occupational domains remains undocumented. Greater evidence is required to promote inclusivity within historically male professions and remove barriers to participation for women and non-binary people in specialised occupations. Representation of non-binary individuals within the literature was not observed, and future research is recommended to be inclusive of all potential workers.

### 3.3 Personal Protective Equipment (PPE)

The 16 included publications assessed occupational performance while participants wore a variety of PPE, including gloves (n = 6) [25, 28–30, 32, 34]; body armour (n = 5) [5, 14, 19–21]; spacesuits (n = 3) [26, 27, 31]; firefighting uniforms (n = 1) [12]; and helmets (n = 1) [45]. Interestingly, all military studies in the present review investigated body armour [5, 14, 19–21] and all uniforms were specific to firefighting [12], while gloves were widely assessed in a healthcare context (vinyl exam gloves [30, 34] or latex surgical gloves) [25, 30]; for manufacturing and assembly (commercially available work gloves) [32]; and within the aerospace industry (for integration with spacesuits) [28, 29]. No studies investigating boots [64, 65] or protective eyewear [66, 67] met the inclusion criteria for the present study, primarily due to not assessing fit [65, 68–85] or not evaluating the effect of fit on at least one measure of performance (often studies instead measured pain or injury prevalence) [67, 86–99]. As such, there is a paucity of published literature on the extent to which these PPE items impact occupational performance, as well as the role of PPE fit. These data, however, are essential knowledge for organisations and employers procuring PPE for diverse user populations.

Several additional studies met the bulk of criteria for inclusion, but the garment or item being investigated was ultimately not deemed PPE. This included studies investigating performance while participants wore a backpack or some form of load carriage system [100, 101]. As PPE is often designed to be worn with other pieces of equipment, such as backpacks, rifles, and firehoses, it is important to consider the fit of PPE when worn or used with other relevant equipment items. For example, ensuring that a backpack and body armour system are integrated well enough to minimise further performance detriments is important for the dismounted soldier. Research has shown that poor sized PPE is associated with increased integration issues [20, 102], which has also been linked to a decreased ability to complete occupation-specific tasks [20]. As the physiological burden of PPE differs based on the equipment with which it is being used [103], future studies should consider evaluating performance while participants wear all relevant equipment items, such as a backpack or load carriage system, in combination with PPE, such as body armour, firefighting uniforms, or helmets.

### 3.4 Fit of PPE

#### 3.4.1 Fit conditions & sizing

As a criterion for inclusion in the present review, all 16 studies made a functional performance-based comparison between at least two and as many as five different fit conditions of PPE. Typically, participants wore PPE that was determined to be the correct size (based on the assessment described in Section 3.4.2) and several other sizes that were deemed to be too small or too large [5, 14, 21, 25, 27–30, 32, 34]. Additional studies also compared performance while participants wore PPE that fit certain body dimensions and not others (e.g. PPE fits chest but not stature compared to stature but not chest) [19, 31] or compared performance between participants whose current PPE was subjectively assessed as a good fit or a poor fit [12, 20, 26, 45].

In a majority of studies, however, this methodology is inherently biased, in that it restricts fit conditions to the available PPE sizing systems, which may not effectively accommodate the user population [11, 13, 20, 23, 24]. For example, body armour is often issued in unisex sizes, which does not provide adequate allowance for differences in size, shape, or position of breast tissue, thus preventing many female soldiers from being able to achieve correct fit in any available size [13, 20, 23, 104]. Similarly, females have smaller hands but are issued the same size and design of firefighting glove, which has been associated with reduced dexterity and occupational performance detriments [105, 106]. Current methodologies do not account for limitations of the existing sizing range, which limits design guidance for future PPE. Future research should consider three-dimensional body size and shape dimensions and their multivariate interactions for PPE design.

#### 3.4.2 Initial size assessment for fit conditions

Investigating the role of PPE fit on occupational performance necessitates a baseline fit condition, which is typically assumed to be “best fit” and compared against sizes that are larger and/or smaller. However, the determination of initial sizing is often biased, in that it relies upon highly subjective opinions and criteria. Several studies in the present review determined initial fit condition via participant selection (i.e. the participant chose their own best-fit PPE; n = 4) [20, 25, 30, 34], which is limited by the lack of standardization and objectivity in assessment. Perceived fit (and therefore size selection) is greatly dependent upon personal preference (e.g. preference for a tighter fit) and individual comfort (e.g. more comfortable in larger size) [18], which can vary widely between participants within the same “fit” condition. Additional studies determined initial fit of PPE through anthropometry and sizing charts (i.e. researchers used measurements and/or a predetermined sizing chart to determine PPE size for each participant; n = 5) [19, 27–29, 32], which limits potential fit to the existing sizing range. Studies also determined initial sizing by visual inspection (i.e. researchers or subject matter experts visually confirmed fit of the PPE for each participant based on prior experience or criterion; n = 4) [21, 26, 31, 45] or a combination of anthropometry and visual inspection (n = 2) [5, 14]. However, there is often limited standardisation in determining sizing between these subject matter experts due to a lack of objective, validated criteria. One included study asked participants to wear their own firefighting pants and quantified perceptions of fit by (a) subjective evaluations via a survey, (b) 3D body scanning, and (c) exit interviews [12]. Future studies are encouraged to employ some combination of these methods, whereby the initial size is determined by quantitative three-dimensional anthropometric shape measures and then confirmed or possibly modified by subject matter expert based on objective criteria as being the best fit of the available sizing range. The initial sizing step is crucial, as no valid comparison of fit will be possible if the initial sizing is not done in a standardised manner.

#### 3.4.3 Static fit evaluation

A minority of studies evaluated static fit, or how the wearer’s anthropometry corresponds to the system’s geometry in a standardised posture after the initial sizing selection (n = 3) [12, 19, 20]. One method for assessing static fit is measuring one to two standard anthropometric dimensions of the individual either via anthropometric tools such as tape measures, callipers, and anthropometers, or extracted from three-dimensional scans to provide information about the geometry of the individual’s body [18]. These measurements are then compared to the dimensions of the system to make a determination of static fit. However, it is important to consider what relative sizing between the user population and PPE is appropriate, and to then use these data to define objective criteria for fit evaluations of each specific PPE item. Even when such criteria have been developed, consideration must be given to the relative weighting between criteria. For example, bra fit studies traditionally determine fit using established pass/fail criteria [107–109] and an overall fail may be associated with a relatively minor fit issue, but is indistinguishable from an overall fail associated with several major fit issues. Without specificity, the resultant fit data is insufficient to inform design changes or sizing alteration. Advancements in three-dimensional body scanning enable organisations to collect numerable anthropometric measures that can inform static fit with greater specificity. It is recommended to incorporate three-dimensional anthropometry and shape dimensions (in functionally-relevant postures and operational tasks) to quantify the interactions between the user and the PPE. Researchers also need to consider how they quantify and evaluate static fit after initial sizing has been performed. Validated, objective assessment criteria must be developed and utilised to improve the consistency of visual and/or virtual inspection. These data can also help characterise and disambiguate preferences and perception-based fit approaches.

#### 3.4.4 Dynamic fit evaluation

All studies included in the present review assessed dynamic fit (how the wearer and the system interact during functional ROM and task performance; n = 16) [5, 12, 14, 19–21, 25–32, 34, 45]. Dynamic fit is important to assess in the context of occupation specific tasks, as the aim of the equipment item should be to minimize restrictions on mobility, and associated fatigue, metabolic cost, performance, and injury detriments. Commonly, dynamic fit is assessed through the use of ROM and functional task performance, including occupationally relevant tasks [13, 18, 110–112] with standardised ROM tasks and procedures having been developed for some equipment items, such as body armour [15]. These tasks typically compare encumbered and non-encumbered conditions to establish a baseline for performance measures. Importantly, dynamic fit assessment should quantify the three-dimensional interactions between the wearer and the system during a range of functional poses or occupation-specific tasks while wearing the equipment item. Furthermore, given the long durations in which workers are required to wear PPE [20, 113], evaluation of performance during prolonged field-based exercises has been recommended for ecological validity and to better assess of the effects of fit of PPE on task performance.

#### 3.4.5 Cognitive fit evaluation

Approximately half of studies also included some assessment of cognitive fit, or how the wearer’s integrated perception-cognition-action outcomes are impacted during system wear (n = 9) [5, 20, 25, 27–30, 32, 34]. Research has demonstrated the high relevance of cognitive tasks to occupational settings that require an underlying capacity for sustained attention (e.g. operational/tactical personnel, occupations that require intense concentration such as surgeons) [114, 115]. In such occupations, the user’s cognitive capabilities must be maintained such that operational or task performance is unfettered. Cognitive fit can be assessed indirectly through an occupational task that requires information processing, such as perception, attention, memory, or problem-solving, or assessed directly through a specific task designed to evaluate a particular cognitive construct. The indirect approach is more widely represented by the studies included in this review; 80% of the 10 publications that assessed cognitive fit did so through an occupational task that naturally involved the perception-cognition-action decision process rather than through a task that directly measures an individual construct. A subset of publications (n = 2) also included direct measures of constructs, such as perception thresholds [29, 30]. The operational tasks ranged from marksmanship [5] to timed performance of a manual task (e.g. dexterity, tactility, accuracy) [25, 28–30, 34]. Alternatively, studies might employ a cognitive task to directly assess an individual construct, such as how PPE influences attention, problem-solving, or motion inhibition. A range of cognitive tasks from the field of psychology have been adopted in other performance-based literature, including sporting, aging, and military settings to evaluate cognitive performance. Exemplary tasks that may be suitable for future studies include the psychomotor vigilance task (assesses vigilance and response speed) [114, 116–118], n-back task (assesses working memory) [119, 120], Task-Switching (assesses cognitive flexibility) [121], and the sustained attention to response task (assesses sustained attention and response inhibition) [122].

### 3.5 Methodological quality

When the methodological quality of each study was critically appraised based on the Mixed Methods Appraisal Tool criteria (MMAT; Table 3), a minority were considered to have a strong overall methodological design (indicated by a score of 2 for each checklist item) [44]. Indeed, only six articles had a low risk of bias across all scoring domains [14, 19–21, 25, 32] and the remaining ten articles scored ‘unclear’ or ‘high’ risk of bias across one or more domains. Specifically, four studies scored ‘high risk’ due to concerns that the participants were not representative of the target population (Item 3^**†**^; either university students or adults who met eligibility criteria without any specific experience) [26, 28–30]. Another three studies scored ‘unclear risk’ for this same checklist item. One study did not clearly describe whether the participants worked in an area that would require +Gz endurance/tolerance [27]; another used participants from the ‘acceleration subject panel’ and it was unclear if they were representative of the user population (pilots) or if they were simply cleared to participate in the research [45]; a third study used ‘experienced centrifuge subjects’, but did not specify whether these were military aircrew or past research participants [31].

Similarly, three publications failed to account for confounding variables in the study design and analysis (Item 6^**†**^). One of these studies assessed the effect marksmanship, but did not control for the shooting ability of the participants in the study [5]; the second study assessed the fit of gloves by participants selecting their preferred size, but did not assess how the selected gloves fit or participant hand anthropometry [34]; and the third study did not standardise fit across the participants [30]. Another study was deemed to have an ‘unclear risk’ in terms of the measurements being appropriate for the outcome and intervention (Item 4^**†**^), such that it was not specified if the questionnaire used in the study was validated [19].

Of note, one particular study scored poorly in a number of categories [12]. Specifically, this study was deemed to be at ‘high risk’ for its qualitative data collection methods, as the exit interviews used may not have been adequate to address the aims of the study (Item 4*); ‘high risk’ for the Item 4^**‡**^, due to the small sample size (n = 18); ‘high’ risk for appropriate statistical analysis to answer the research question (Item 7^**‡**^) as multiple analyses were used without being adjusted for, increasing the chance of error. Similarly, the same study scored ‘high risk’ for the integration of qualitative and quantitative components (Item 5^**§**^), as the discussion was very brief and the integration of data was not deemed to be sufficient; ‘unclear risk’ for divergences and inconsistencies between quantitative and qualitative results, as these were not adequately addressed in the paper (Item 6^**§**^); and ‘high risk’ for different components of the study failing to adhere to the quality criteria of each tradition of the methods involved (Item 7^**§**^), as there was a lack of information regarding how the qualitative data would be analysed.

It is important to note that the MMAT used to rate the methodological quality of these publications did not have an item for participant characteristics.[44] Several studies failed to report where participants were recruited from (general or specialised population) [26]; the age of participants (either altogether [19] or only reported the age range [30, 45]); and even the sex of participants [32], all of which are substantial limitations in interpreting and generalising the study results, but none of which were accounted for in the MMAT quality assessment. A different tool may have more accurately represented the quality of publications by accounting for these missing participant characteristics; however, the MMAT was chosen for its ability to assess both quantitative and qualitative studies (as well as mixed methods studies), all of which appear in the present review.

### 3.6 Performance while wearing PPE

#### 3.6.1 Performance measures

Studies considered a variety of performance measures, broadly classed as static (performance of physical tasks that do not involve motion; e.g. isometric tasks) [19, 30]; dynamic (i.e. performance of physical tasks that involve motion; e.g. range of motion tasks) [5, 12, 14, 19–21, 25–32, 34, 45]; and cognitive (i.e. performance of mental process of perception, learning, memory, understanding, awareness, reasoning, judgement, intuition, and language) [5, 20, 25, 27–30, 32, 34]. Specifically, studies assessed task-specific performance (e.g. time taken to complete an occupational task or marksmanship accuracy; n = 6) [5, 25, 28–30, 34]; subjective fit and comfort (n = 3) [12, 19, 20]; pulmonary and cardiorespiratory function (n = 2) [21, 31]; ROM (n = 2) [14, 26]; endurance (n = 1) [27]; and muscle activation patterns (n = 1) [32].

Generic ROM tasks, which assess dynamic performance, may be insufficient to adequately characterise performance during occupation specific tasks. For example, female soldiers report extreme difficulty assuming prone rifle postures while wearing essential items of PPE, such as body armour and a helmet [113]. As this task involves multiple factors (integration between the helmet and body armour when lifting the head to obtain a sight picture, sufficient strength, and coordination to hold the rifle in position), ROM tasks alone are likely inadequate to characterise performance during this task. Task analysis should be undertaken to determine key operational tasks prior to evaluation of performance in PPE. In some occupational domains, standardised dynamic tasks have already been developed, such as the load effects assessment program (LEAP) used in military settings [123]. In addition, a range of occupation specific tasks have been defined by physical employment standards literature, which are likely to be suitable for research evaluating performance of PPE [124–127]. Future research should consider the effects of the fit of PPE in their evaluations.

Additionally, given the importance of cognitive performance in a range of occupational settings as discussed in Section 3.4.5, future research evaluating human performance in PPE should aim to include cognitive performance measures (either tasks designed specifically to evaluate a cognitive construct or operational assessments that inherently consider cognition). A number of studies have been conducted evaluating cognitive performance during load carriage [119, 128–131], but relatively few studies have examined cognitive performance wearing PPE [132, 133]. Concomitant considerations when wearing PPE, such as heat stress, have been shown to negatively impact cognitive performance [132–134]. As such, incorporating cognitive measures into evaluations of performance while wearing PPE is required to extend the current body of research beyond the physical impacts of PPE on performance. Ensuring that the tasks undertaken in research studies reflect the occupational demands of the users whilst wearing PPE should be a key focus. Importantly, to understand the impacts of PPE on human movement capability, it is necessary to consider the systems that influence the perception-cognition-action cycle. Therefore, research evaluating PPE crosses a range of research fields including physiology, biomechanics, ergonomics, and human factors. A multi-disciplinary approach to evaluating human performance while wearing PPE is recommended in future research.

#### 3.6.2 Effect of PPE fit on performance

The key findings and recommendations regarding the effect of fit of PPE on performance from the 16 studies included in the present review are summarised in Table 4. As one of the key selection criteria, all of the selected studies included fit as an independent variable; in other words, these studies analysed performance differences while participants wore PPE in varying degrees of fit (e.g. “best fit” compared to “too large” or “too small”). A majority (88%; n = 14) [5, 12, 14, 19–21, 25–32] of these studies found that the fit of PPE had a statistically significant effect on occupational performance. Poorly sized PPE resulted in a range of performance detriments, including slower or increased reaction time [5, 25, 28, 30]; decreased ROM or mobility [12, 14, 19, 20, 26]; decreased endurance or tolerance [27, 31]; decreased pulmonary function [21]; and altered muscle activation [32]. Given that PPE wear is associated with a human systems integration and mass burden, the results of these studies highlight that negative performance effects can be amplified if PPE is ill-fitting. However, the fit of PPE is a modifiable factor, which can be addressed by improving the metrics to quantify fit and developing the range of PPE sizing accordingly.

Of note, however, are the two studies that did not observe any effect of PPE fit on occupational performance. Drabek et al. 2013 reported that there was no significant difference in response time on a manual dexterity task (peg-board) when participants wore vinyl examination gloves in their preferred size compared to either too small or too large [34]. However, participants reported some degree of ill fit in all sizes, which suggests either that the design or the existing sizing range (small–extra-large) of vinyl examination gloves used in this study was not adequately catering to the target population. McCloskey and Esken also found that integrated night vision goggle helmet fit had no significant impact upon performance in a human-rated centrifuge [45]. These results suggest that the effects of sizing and associated fit of PPE on performance vary by equipment item and task, and therefore research should seek to evaluate all PPE items required for occupational task performance within each respective occupational domain to prioritise the fit of those equipment items that are most detrimental to task performance.

#### 3.6.3 Effect of PPE on performance

Half of the included studies (n = 8) identified a significant effect of PPE (regardless of fit) on the occupational performance of participants [5, 14, 21, 26, 28–30, 32], while only two studies reported no significant difference between performance when wearing PPE compared to not wearing the PPE item [25, 34]. Both were studies concerned with surgical gloves, and both ultimately reported that use of the selected surgical gloves had no impact on manual dexterity in a healthcare setting when compared to being bare-handed. The finding that PPE affects performance in most studies is not surprising given that PPE is known to impose a mass, bulk, and human systems integration burden [10]. Therefore, strategies to minimise the negative effects of PPE are required. Ensuring PPE is correctly fitted to the anthropometric dimensions of the user is a key strategy to reduce the performance detriments associated with PPE use, as well as promoting dynamic and cognitive fit of the system. The remaining six studies did not assess a baseline condition that allowed for comparison between PPE and non-PPE conditions [12, 19, 20, 27, 31, 45]. Baseline conditions can be used to identify the magnitude of the PPE impact and subsequently design interventions to reduce the impact, or to support supplementary tool design to reduce PPE impacts, and therefore are worthwhile in future research.

### 3.7 Recommendations for future research

Based on the key findings collated and compared across studies included within this review, as well as the gaps identified in the current body of knowledge, the following section outlines recommendations for future research assessing the effect of PPE fit on functional performance across a range of occupational domains.

#### 3.7.1 Occupational domains

Many occupational domains in which PPE is required and potentially detrimental to operational performance are not represented in the present review due to methodological limitations or study design, and therefore the extent to which PPE fit affects performance in these occupations remains unknown. Future studies are encouraged to examine the fit of PPE across a diverse range of occupational domains, including scientific laboratories, construction, mining, surgery, firefighting, policing, and manufacturing and assembly.

#### 3.7.2 Participants and sampling approach

A majority of the studies in this review included fewer than 30 participants and several studies recruited a generic sample population (e.g. university students) from a narrow age range. A diversity of participants is essential to ensure that the results are generalisable to the intended end-user population. Therefore, future studies are recommended to recruit a larger sample size from a broad age range, ensuring that the sample is representative of the user population and that it adequately accounts for human variability (e.g. anthropometry, posture, behaviour, etc). Furthermore, females and non-binary individuals remain underrepresented in literature pertaining to protective equipment, despite a bulk of research indicating that females are disproportionately affected by ill-fit and PPE-related performance detriments. It is therefore imperative that further research be inclusive of all potential workers.

#### 3.7.3 Type of PPE

A range of personal protective equipment is widely used across occupational domains, but no published research has explored the performance detriments associated with fit of boots, protective eyewear, surgical gowns, police body armour, harnesses, exoskeletons, or many other types of PPE. Although they are represented by several studies in the present review, body armour, gloves, helmets, spacesuits, and firefighting uniforms also warrant further investigation in a range of occupational contexts. Future research is encouraged to explore the fit of a range of PPE used in occupational settings, especially considering the fit and integration of related equipment items that may influence PPE fit (e.g. backpacks, oxygen cylinders, weapons, etc). Furthermore, to better cater to the diverse anthropometry of worker populations, future research should endeavour to examine greater ranges of sizes and designs within assessable equipment items. This includes exploring mass customisation and custom PPE approaches to evaluating the effect of PPE on performance in occupational settings.

#### 3.7.4 PPE initial size assessments for fit conditions

In order to determine initial sizing, which is often used as the “best fit” condition within PPE research, future studies are encouraged to employ some combination of participant self-selection, visual inspection by a subject matter expert, and objective criteria to determine initial size, whereby participants self-select a size or quantitative three-dimensional anthropometric shape measures are used to select a size that is then confirmed or possibly modified by the subject matter expert based on standardised criteria to determine the best possible fit from the available sizing range. The specific methods used for initial PPE sizing should be informed by the unique goals of each project; that is, operations affected by comfort of the user within the system will benefit from the inclusion of subjective feedback (i.e. participant self-selection) while research concerned with the specific relation of PPE fit to the user dimensions should rely predominantly on objective anthropometric data.

#### 3.7.5 Fit evaluation

In evaluating the fit of PPE equipment items, there is a need for further objectivity and standardisation. Future research should also aim to incorporate a combination of **s**tatic, dynamic, and cognitive assessments of fit, as all three categories have the potential to affect occupational task performance. Specific recommendations for assessing fit within each category are included in the following three sections.

#### 3.7.6 Static fit

It is recommended that future static fit assessments incorporate three-dimensional anthropometry in functional postures relevant to operational tasks, as this will yield a more robust quantification of the interactions between the human body and PPE item/system. Visual inspection by a subject matter expert or subjective user feedback may be used as additional tools to achieve static fit within the PPE item, but it is further recommended that a validated assessment criteria be developed and utilised to improve any static fit assessment. Criteria may also consider user preferences and perception-based fit approaches.

#### 3.7.7 Dynamic fit

Although they have been widely employed in the current literature, dynamic fit assessments can be improved to quantify the three-dimensional interactions more directly between the wearer and the equipment item during a range of functional movements and occupation-specific tasks. Importantly, evaluation of performance during prolonged field-based exercises is recommended to ensure ecological validity of study results and to assess of the effects of dynamic fit of PPE on in-situ task performance.

#### 3.7.8 Cognitive fit

It is recommended that future research include cognitive fit assessments as part of the performance evaluation, which can include indirect assessment through an occupational task that requires perception, attention, memory, and/or problem-solving or direct assessment through a specific task designed to evaluate a particular cognitive construct. A range of cognitive tasks from the field of psychology have been adopted in other performance-based literature, including sporting, aging, and military settings to evaluate cognitive performance and provide exemplar cognitive tasks that can be adopted in future research examining cognitive fit.

#### 3.7.9 Performance while wearing PPE

In order to quantify the effect of PPE fit on performance, key tasks relative to the occupational role should first be undertaken without the equipment item to determine a baseline (i.e. a standard towards which PPE fit and design improvements should aim). With a baseline measurement established, the magnitude of the performance decrement or enhancement when wearing PPE can be determined. The methods by which studies assess user performance while wearing PPE should be specific to the occupational domain and simulate operational conditions where possible. Researchers are also encouraged to work towards standardised tasks (e.g. the load effects assessment program (LEAP) used in military settings), as this will facilitate comparison between studies and a greater understanding of the role of PPE fit on occupational performance detriments.

### 3.8 Limitations of the review

This review is not without limitations. Although a hand search was conducted of the International Conference on Environmental Systems congress proceeding database to identify additional published articles, other grey literature were not included, potentially introducing publication bias and omitting relevant evidence [135, 136]. As discussed within the main body of this review, the results suggest the need for new and confirmatory studies that recruit a more representative population; consider sex as a covariate; evaluate PPE fit and performance when integrated with all relevant equipment items; include outcome measures related to all three categories of fit (static, dynamic, cognitive); and assess performance of operationally-relevant tasks.

## 4 Conclusion

Across occupational domains, previous research has evaluated the effect of PPE on various types of human performance; however, few studies have considered the role of fit. Of the 16 studies in this review, 88% reported that the fit of PPE had a statistically significant effect on occupational performance. Poorly sized PPE was associated with range of performance detriments, including slower reaction time; decreased ROM or mobility; decreased endurance or tolerance; decreased pulmonary function; and altered muscle activation. However, limited research met the inclusion criteria for this review, which suggests gaps in the current understanding of the impact of PPE fit on operational performance across a range of occupational domains. The included publications had a high risk of overall bias based on methodology quality. Future research should aim to recruit a more representative population; consider sex as a covariate; quantify and evaluate PPE fit and performance when integrated with all relevant equipment items; include outcome measures related to all three categories of fit (static, dynamic, cognitive); and assess performance of operationally relevant tasks.

## Supporting information

S1 Checklist PRISMA 2020 checklist. (DOCX)

## References

[1] Safe Work Australia. "Personal protective equipment." https://www.safeworkaustralia.gov.au/ppe (accessed 1 June 2021.

[2] Berger M. A., Krul A. J., and Daanen H. A., "Task specificity of finger dexterity tests," *Applied ergonomics*, vol. 40, no. 1, pp. 145–147, 2009. doi: 10.1016/j.apergo.2008.01.014 18339353

[3] Chang C.-H., Wang M.-J. J., and Lin S.-C., "Evaluating the effects of wearing gloves and wrist support on hand–arm response while operating an in-line pneumatic screwdriver," *International Journal of Industrial Ergonomics*, vol. 24, no. 5, pp. 473–481, 1999.

[4] Freivalds A., "Ergonomics of hand tools," in *Occupational Ergonomics*: *Principles of Work Design*: CRC Press, 2003, pp. 27-1-27-27.

[5] Choi H. J., Mitchell K. B., Garlie T., McNamara J., Hennessy E., and Carson J., "Effects of body armor fit on marksmanship performance," in *Advances in Physical Ergonomics and Human Factors*: Springer, 2016, pp. 341–354.

[6] Laing S. and Jaffrey M., "Thoraco-abdominal Organ Locations: Variations Due to Breathing and Posture and Implications for Body Armour Coverage Assessments," 2019.

[7] NASA. "What Is a Spacesuit?" https://www.nasa.gov/audience/forstudents/5-8/features/nasa-knows/what-is-a-spacesuit-58.html (accessed 6 Jul 2021.

[8] CDC, "Hierarchy of Controls," *The National Institute for Occupational Safety and Health* (NIOSH), 2015.

[9] Watson C., Troynikov O., and Lingard H., "Design considerations for low-level risk personal protective clothing: a review," *Industrial health*, vol. 57, no. 3, pp. 306–325, 2019. doi: 10.2486/indhealth.2018-0040 30089764 PMC6546585

[10] Knapik J. J., Reynolds K. L., and Harman E., "Soldier load carriage: historical, physiological, biomechanical, and medical aspects," *Military medicine*, vol. 169, no. 1, pp. 45–56, 2004. doi: 10.7205/milmed.169.1.45 14964502

[11] McQuerry M., "Effect of structural turnout suit fit on female versus male firefighter range of motion," *Applied Ergonomics*, vol. 82, p. 102974, 2020. doi: 10.1016/j.apergo.2019.102974 31606711

[12] Park J. and Langseth-Schmidt K., "Anthropometric fit evaluation of firefighters’ uniform pants: A sex comparison," *International Journal of Industrial Ergonomics*, vol. 56, pp. 1–8, 2016.

[13] Coltman C. E., Brisbine B. R., Molloy R. H., and Steele J. R., "Can smaller body armour improve thoracolumbar range of motion and reduce interference when female soldiers perform dynamic tasks?," *Applied Ergonomics*, vol. [online ahead of print], 2021. 10.1016/j.apergo.2021.103602 34662749

[14] H. J. Choi, K. B. Mitchell, T. N. Garlie, and L. DeSimone, "Effects of body armor fit on Warfighter mobility as measured by range of motion (ROM)," in *International Conference on Applied Human Factors and Ergonomics*, 2018: Springer, pp. 16–28.

[15] Mitchell K. B., "Standard methodology for assessment of range of motion while wearing body armor," ARMY NATICK SOLDIER RESEARCH DEVELOPMENT AND ENGINEERING CENTER MA, 2013.

[16] Holmer I., "Protective clothing in hot environments," *Industrial health*, vol. 44, no. 3, pp. 404–413, 2006. doi: 10.2486/indhealth.44.404 16922184

[17] Hostler D. et al., "The effect of hyperhydration on physiological and perceived strain during treadmill exercise in personal protective equipment," *European journal of applied physiology*, vol. 105, no. 4, pp. 607–613, 2009. doi: 10.1007/s00421-008-0940-2 19037655

[18] Stirling L. et al., "Static, dynamic, and cognitive fit of exosystems for the human operator," *Human factors*, vol. 62, no. 3, pp. 424–440, 2020. doi: 10.1177/0018720819896898 32004106

[19] Davis J. I., Lewis E., and Ellett J., "A fit and function analysis of the UK OSPREY body armour system for female users," *BMJ Military Health*, 2020. doi: 10.1136/jramc-2019-001248 32098901

[20] Coltman C. E., Steele J. R., Spratford W. A., and Molloy R. H., "Are female soldiers satisfied with the fit and function of body armour?," *Applied Ergonomics*, vol. 89, p. 103197, 2020. doi: 10.1016/j.apergo.2020.103197 32755739

[21] Armstrong N. C., Ward A., Lomax M., Tipton M. J., and House J. R., "Wearing body armour and backpack loads increase the likelihood of expiratory flow limitation and respiratory muscle fatigue during marching," *Ergonomics*, vol. 62, no. 9, pp. 1181–1192, 2019. doi: 10.1080/00140139.2019.1629638 31364962

[22] Hesse S., Werner C., Matthias K., Stephen K., and Berteanu M., "Non–velocity-related effects of a rigid double-stopped ankle-foot orthosis on gait and lower limb muscle activity of hemiparetic subjects with an equinovarus deformity," *Stroke*, vol. 30, no. 9, pp. 1855–1861, 1999. doi: 10.1161/01.str.30.9.1855 10471436

[23] Coltman C. E., Brisbine B. R., and Steele J. R., "Bra-body armour integration, breast discomfort and breast injury associated with wearing body armour," *Ergonomics*, pp. 1–11, 2021. doi: 10.1080/00140139.2021.1951849 34236015

[24] Ciesielska-Wrobel I., DenHartog E., and Barker R., "The influence of designs of protective uniforms on firefighters’ performance during moderate physical exercises," *Textile Research Journal*, vol. 88, no. 17, pp. 1979–1991, 2018.

[25] Drabek T., Boucek C. D., and Buffington C. W., "Wearing the wrong size latex surgical gloves impairs manual dexterity," *Journal of Occupational and Environmental Hygiene*, vol. 7, no. 3, pp. 152–155, 2010. doi: 10.1080/15459620903481660 20017056

[26] Fineman R. A., McGrath T. M., Kelty-Stephen D. G., Abercromby A. F., and Stirling L. A., "Objective metrics quantifying fit and performance in spacesuit assemblies," *Aerospace Medicine and Human Performance*, vol. 89, no. 11, pp. 985–995, 2018. doi: 10.3357/AMHP.5123.2018 30352651

[27] Hearon C., Fischer M., and Dooley J., "Male/female SACM endurance comparison: support for the Armstrong Laboratory modifications to the CSU-13B/P anti-g suit," *Aviation*, *Space*, *and Environmental Medicine*, vol. 69, no. 12, pp. 1141–1145, 1998. 9856537

[28] S. Lombardo, S. Jacobs, K. Duda, and L. Stirling, "Evaluating the Effect of Spacesuit Glove Fit on Dexterity and Cognitive Task Performance," 2020: 2020 International Conference on Environmental Systems.

[29] S. Lombardo, K. Duda, and L. Stirling, "Evaluating the Effect of Spacesuit Glove Fit on Functional Tactility Task Performance," in *2020 IEEE Aerospace Conference*, 2020: IEEE, pp. 1–10.

[30] Mylon P., Lewis R., Carré M. J., and Martin N., "An evaluation of dexterity and cutaneous sensibility tests for use with medical gloves," *Proceedings of the Institution of Mechanical Engineers*, *Part C*: *Journal of Mechanical Engineering Science*, vol. 230, no. 16, pp. 2896–2912, 2016.

[31] Stevenson A. T., Lythgoe D. T., Darby C. L., Devlin J. M., Connolly D. M., and Scott J. P., "Garment fit and protection from sustained+ Gz acceleration with ‘full-coverage’anti-G trousers," *Aviation*, *Space*, *and Environmental Medicine*, vol. 84, no. 6, pp. 600–607, 2013. doi: 10.3357/asem.3487.2013 23745288

[32] Yoo I.-G., Lee J., Jung M.-Y., and Lee J.-H., "Effects of wearing the wrong glove size on shoulder and forearm muscle activities during simulated assembly work," *Industrial Health*, pp. 1107280082–1107280082, 2011. doi: 10.2486/indhealth.ms1235 21804270

[33] Choi H. J., Zehner G. F., and Hudson J. A., "A manual for the performance of protective equipment fit-mapping," OAK RIDGE INST FOR SCIENCE AND EDUCATION WRIGHT-PATTERSON AFB OH, 2009.

[34] Drabek T., Boucek C. D., and Buffington C. W., "Wearing ambidextrous vinyl gloves does not impair manual dexterity," *Journal of Occupational and Environmental Hygiene*, vol. 10, no. 6, pp. 307–311, 2013. doi: 10.1080/15459624.2013.777293 23548060

[35] Choi H. J., Zehner G. F., and Metzger T., "F-35 protective equipment fit assessment: Light weight Coverall," OAK RIDGE INST FOR SCIENCE AND EDUCATION WRIGHT-PATTERSON AFB OH, 2011.

[36] Hsiao H., Whitestone J., and Kau T.-Y., "Evaluation of fall arrest harness sizing schemes," *Human factors*, vol. 49, no. 3, pp. 447–464, 2007. doi: 10.1518/001872007X200094 17552309

[37] Armstrong N. C. and Gay L. A., "The effect of flexible body armour on pulmonary function," *Ergonomics*, vol. 59, no. 5, pp. 692–696, 2016. doi: 10.1080/00140139.2015.1084052 26548548

[38] Chiou S. S., Turner N., Zwiener J., Weaver D. L., and Haskell W. E., "Effect of boot weight and sole flexibility on gait and physiological responses of firefighters in stepping over obstacles," *Human Factors*, vol. 54, no. 3, pp. 373–386, 2012. doi: 10.1177/0018720811433464 22768640

[39] Dianat I., Haslegrave C. M., and Stedmon A. W., "Short and longer duration effects of protective gloves on hand performance capabilities and subjective assessments in a screw-driving task," *Ergonomics*, vol. 53, no. 12, pp. 1468–1483, 2010. doi: 10.1080/00140139.2010.528453 21108084

[40] Dianat I., Haslegrave C. M., and Stedmon A. W., "Using pliers in assembly work: Short and long task duration effects of gloves on hand performance capabilities and subjective assessments of discomfort and ease of tool manipulation," *Applied Ergonomics*, vol. 43, no. 2, pp. 413–423, 2012. doi: 10.1016/j.apergo.2011.06.016 21777904

[41] Lenton G. K., Doyle T. L., Saxby D. J., Billing D., Higgs J., and Lloyd D. G., "Integrating a hip belt with body armour reduces the magnitude and changes the location of shoulder pressure and perceived discomfort in soldiers," *Ergonomics*, vol. 61, no. 4, pp. 566–575, 2018. doi: 10.1080/00140139.2017.1381278 28918698

[42] Zhang L., "Cognitive performance and physiological changes in females at high G while protected with COMBAT EDGE and ATAGS," *Aviation*, *Space*, *and Environmental Medicine*, vol. 70, no. 9, pp. 857–862, 1999. 10503749

[43] Moher D., Liberati A., Tetzlaff J., Altman D. G., and Group P., "Preferred reporting items for systematic reviews and meta-analyses: the PRISMA statement," *PLoS* *medicine*, vol. 6, no. 7, p. e1000097, 2009. 10.1371/journal.pmed.1000097 PMC2707599 19621072

[44] Hong Q. N. et al., "The Mixed Methods Appraisal Tool (MMAT) version 2018 for information professionals and researchers," *Education for Information*, vol. 34, no. 4, pp. 285–291, 2018.

[45] McCloskey K. and Esken R. L., "Evaluation of integrated night vision goggle (NVG) helmets under sustained+ Gz," *Aviation*, *Space*, *and Environmental Medicine*, 1995. 7726774

[46] Villano J. S., Follo J. M., Chappell M. G., and Collins M. T. Jr, "Personal protective equipment in animal research," *Comparative medicine*, vol. 67, no. 3, pp. 203–214, 2017. 28662749 PMC5482512

[47] Hall S. K., *Chemical safety in the laboratory*. CRC Press, 2018.

[48] Akbar‐Khanzadeh F. and Pulido E. V., "Using respirators and goggles to control exposure to air pollutants in an anatomy laboratory," *American Journal of Industrial Medicine*, vol. 43, no. 3, pp. 326–331, 2003. doi: 10.1002/ajim.10180 12594780

[49] Ammad S. et al., "Personal Protective Equipment In Construction, Accidents Involved In Construction Infrastructure Projects," *Solid State Technology*, vol. 63, no. 6, pp. 4147–4159, 2020.

[50] Dobson J. A., Riddiford-Harland D. L., Bell A. F., and Steele J. R., "Are underground coal miners satisfied with their work boots?," *Applied ergonomics*, vol. 66, pp. 98–104, 2018. doi: 10.1016/j.apergo.2017.08.009 28958436

[51] Carpenter W. S., Lee B. C., Gunderson P. D., and Stueland D. T., "Assessment of personal protective equipment use among Midwestern farmers," *American journal of industrial medicine*, vol. 42, no. 3, pp. 236–247, 2002. doi: 10.1002/ajim.10103 12210692

[52] Cappelen A. W., Nygaard K., Sørensen E. Ø., and Tungodden B., "Social preferences in the lab: A comparison of students and a representative population," *The Scandinavian Journal of Economics*, vol. 117, no. 4, pp. 1306–1326, 2015.

[53] Kirk N. and Ridgway S., "Ergonomics testing of consumer products 1. General considerations," *Applied ergonomics*, vol. 1, no. 5, pp. 295–300, 1970. doi: 10.1016/0003-6870(70)90080-3 15676342

[54] Wilson J. R., "Participation—A framework and a foundation for ergonomics?," *Journal of occupational psychology*, vol. 64, no. 1, pp. 67–80, 1991.

[55] Bennett A., Hanley J., Buckle P., and Bridger R., "Work demands during firefighting training: does age matter?," *Ergonomics*, vol. 54, no. 6, pp. 555–564, 2011.

[56] Abt J. P., Perlsweig K., Nagai T., Sell T. C., Wirt M. D., and Lephart S. M., "Effects of age and military service on strength and physiological characteristics of US Army soldiers," *Military medicine*, vol. 181, no. 2, pp. 173–179, 2016. 26837087 10.7205/MILMED-D-15-00036

[57] Kenny G. P., Groeller H., McGinn R., and Flouris A. D., "Age, human performance, and physical employment standards," *Applied physiology*, *nutrition*, *and metabolism*, vol. 41, no. 6, pp. S92–S107, 2016. 10.1139/apnm-2015-0483 27277571

[58] Bryden P. and Roy E., "A new method of administering the Grooved Pegboard Test: performance as a function of handedness and sex," *Brain and cognition*, vol. 58, no. 3, pp. 258–268, 2005. doi: 10.1016/j.bandc.2004.12.004 15963376

[59] M. FISCHER, J. WIEGMAN, and D. BAUER, "Female tolerance to sustained acceleration- A retrospective study," in *Annual SAFE Symposium, 29 th, Las Vegas, NV*, 1992, pp. 283–287.

[60] Edwards M., Furnell A., Coleman J., and Davis S., "A preliminary anthropometry standard for Australian Army equipment evaluation," *Land Division*, *Defence Science and Technology Organisation*, 2014.

[61] McLellan T. M., "Sex-related differences in thermoregulatory responses while wearing protective clothing," *European journal of applied physiology and occupational physiology*, vol. 78, no. 1, pp. 28–37, 1998. doi: 10.1007/s004210050383 9660153

[62] Shapiro Y., Pandolf K. B., Avellini B. A., Pimental N. A., and Goldman R. F., "Physiological responses of men and women to humid and dry heat," *Journal of Applied Physiology*, vol. 49, no. 1, pp. 1–8, 1980. doi: 10.1152/jappl.1980.49.1.1 7399982

[63] Watkins E. R., Walker A., Mol E., Jahnke S., and Richardson A. J., "Women firefighters’ health and well-being: an international survey," *Women’s Health Issues*, vol. 29, no. 5, pp. 424–431, 2019. doi: 10.1016/j.whi.2019.02.003 30930075

[64] Dobson J. A., Riddiford-Harland D. L., Bell A. F., and Steele J. R., "Effect of work boot type on work footwear habits, lower limb pain and perceptions of work boot fit and comfort in underground coal miners," *Applied ergonomics*, vol. 60, pp. 146–153, 2017. doi: 10.1016/j.apergo.2016.11.008 28166873

[65] Rawcliffe A. J. et al., "The Effects of British Army Footwear on Ground Reaction Force and Temporal Parameters of British Army Foot Drill," *The Journal of Strength & Conditioning Research*, vol. 34, no. 3, pp. 754–762, 2020. doi: 10.1519/JSC.0000000000002139 28800005

[66] Harrison M. F., Coffey B., Albert W. J., and Fischer S. L., "Night vision goggle-induced neck pain in military helicopter aircrew: a literature review," *Aviation*, *space*, *and environmental medicine*, vol. 86, no. 1, pp. 46–55, 2015. doi: 10.3357/AMHP.4027.2015 25565533

[67] Lombardi D. A., Verma S. K., Brennan M. J., and Perry M. J., "Factors influencing worker use of personal protective eyewear," *Accident Analysis & Prevention*, vol. 41, no. 4, pp. 755–762, 2009. doi: 10.1016/j.aap.2009.03.017 19540964

[68] Kesler R. M. et al., "Impact of SCBA size and fatigue from different firefighting work cycles on firefighter gait," *Ergonomics*, vol. 61, no. 9, pp. 1208–1215, 2018. doi: 10.1080/00140139.2018.1450999 29569521

[69] Wiszomirska I., Iwańska D., Tabor P., Karczewska-Lindinger M., Urbanik C., and Mastalerz A., "Postural stability pattern as an important safety factor of firefighters," *Work*, vol. 62, no. 3, pp. 469–476, 2019. doi: 10.3233/WOR-192881 30909262

[70] Donoghue A. J. et al., "Impact of personal protective equipment on pediatric cardiopulmonary resuscitation performance: a controlled trial," *Pediatric emergency care*, vol. 36, no. 6, p. 267, 2020. doi: 10.1097/PEC.0000000000002109 32483079 PMC7274141

[71] White S. C. and Hostler D., "The effect of firefighter protective garments, self-contained breathing apparatus and exertion in the heat on postural sway," *Ergonomics*, vol. 60, no. 8, pp. 1137–1145, 2017. doi: 10.1080/00140139.2016.1257162 27822982

[72] Park K., Hur P., Rosengren K. S., Horn G. P., and Hsiao-Wecksler E. T., "Effect of load carriage on gait due to firefighting air bottle configuration," *Ergonomics*, vol. 53, no. 7, pp. 882–891, 2010. doi: 10.1080/00140139.2010.489962 20582769

[73] Vorih D. C., Bolton L. D., Marcelynas J., Nowicki T. A., Jacobs L., and Robinson K. J., "Comparison of nitrile gloves and nitrile over nomex gloves," *Air medical journal*, vol. 28, no. 6, pp. 288–302, 2009. doi: 10.1016/j.amj.2009.06.001 19896579

[74] Willson D. et al., "The performance of field scientists undertaking observations of early life fossils while in simulated space suit," *Acta Astronautica*, vol. 93, pp. 193–206, 2014.

[75] Coca A., Roberge R., Shepherd A., Powell J., Stull J., and Williams W., "Ergonomic comparison of a chem/bio prototype firefighter ensemble and a standard ensemble," *European Journal of Applied Physiology*, vol. 104, no. 2, pp. 351–359, 2008. doi: 10.1007/s00421-007-0644-z 18075754

[76] Heller M. F., Challis J. H., and Sharkey N. A., "Changes in postural sway as a consequence of wearing a military backpack," *Gait & posture*, vol. 30, no. 1, pp. 115–117, 2009. 19403310 10.1016/j.gaitpost.2009.02.015

[77] Kesler R. M. et al., "Impact of SCBA size and firefighting work cycle on firefighter functional balance," *Applied ergonomics*, vol. 69, pp. 112–119, 2018. doi: 10.1016/j.apergo.2018.01.006 29477318

[78] Chander H. et al., "Impact of military type footwear and workload on heel contact dynamics during slip events," *International journal of industrial ergonomics*, vol. 66, pp. 18–25, 2018.

[79] Park K., Rosengren K. S., Horn G. P., Smith D. L., and Hsiao-Wecksler E. T., "Assessing gait changes in firefighters due to fatigue and protective clothing," *Safety science*, vol. 49, no. 5, pp. 719–726, 2011.

[80] Coates M. J., Jundi A. S., and James M. R., "Chemical protective clothing; a study into the ability of staff to perform lifesaving procedures," *Emergency Medicine Journal*, vol. 17, no. 2, pp. 115–118, 2000. 10.1136/emj.17.2.115 PMC1725348 10718233

[81] Moser D. J., Graham R. B., Stevenson J. M., and Costigan P. A., "Subjective and objective analysis of three water pump systems carried by forest firefighters," *Work*, vol. 47, no. 1, pp. 45–53, 2014. doi: 10.3233/WOR-131690 24004749

[82] Coyne K. M. and Barker D. J., "Speech intelligibility while wearing full-facepiece air-purifying respirators," *Journal of occupational and environmental hygiene*, vol. 11, no. 11, pp. 751–756, 2014. doi: 10.1080/15459624.2014.908257 24689436

[83] Chander H., Garner J. C., and Wade C., "Slip outcomes in firefighters: A comparison of rubber and leather boots," *Occupational Ergonomics*, vol. 13, no. 2, pp. 67–77, 2016.

[84] Chander H. et al., "Impact of military type footwear and load carrying workload on postural stability," *Ergonomics*, vol. 62, no. 1, pp. 103–114, 2019. doi: 10.1080/00140139.2018.1521528 30196761

[85] Rosengren K. S., Hsiao-Wecksler E. T., and Horn G., "Fighting fires without falling: Effects of equipment design and fatigue on firefighter’s balance and gait," *Ecological Psychology*, vol. 26, no. 1–2, pp. 167–175, 2014.

[86] D. Belakova, I. Dabolina, I. Baltina, and G. Zommere, "Improvement of Workwear Clothing for Army," in *IOP Conference Series: Materials Science and Engineering*, 2017, vol. 254, no. 15: IOP Publishing, p. 152002.

[87] M. M. White, D. B. Kaber, Y. Deng, and X. Xu, "Design Process for an Ergonomic Solution to the Police Duty Belt," in *International Conference on Applied Human Factors and Ergonomics*, 2018: Springer, pp. 3–15.

[88] Harrod M. et al., "Understanding workflow and personal protective equipment challenges across different healthcare personnel roles," *Clinical Infectious Diseases*, vol. 69, no. Supplement_3, pp. S185–S191, 2019. doi: 10.1093/cid/ciz527 31517971

[89] E. W. Obropta and D. J. Newman, "A comparison of human skin strain fields of the elbow joint for mechanical counter pressure space suit development," in *2015 IEEE Aerospace Conference*, 2015: IEEE, pp. 1–9.

[90] Mylon P., Lewis R., Carré M. J., Martin N., and Brown S., "A study of clinicians’ views on medical gloves and their effect on manual performance," *American journal of infection control*, vol. 42, no. 1, pp. 48–54, 2014. doi: 10.1016/j.ajic.2013.07.009 24268835

[91] Lee H., Hong K. H., Kim S., and Lee Y., "Effects of fit on pressure distribution and momentum of ballistic body armor vest in jump," *Textile Research Journal*, vol. 83, no. 14, pp. 1514–1523, 2013.

[92] Lee W., Yang X., Jung D., Park S., Kim H., and You H., "Ergonomic evaluation of pilot oxygen mask designs," *Applied ergonomics*, vol. 67, pp. 133–141, 2018. doi: 10.1016/j.apergo.2017.10.003 29122184

[93] Van den Oord M. H., Steinman Y., Sluiter J. K., and Frings-Dresen M. H., "The effect of an optimised helmet fit on neck load and neck pain during military helicopter flights," *Applied ergonomics*, vol. 43, no. 5, pp. 958–964, 2012. doi: 10.1016/j.apergo.2012.01.004 22356840

[94] Van den Oord M. H., Frings-Dresen M. H., and Sluiter J. K., "Optimal helmet use and adjustments with respect to neck load: The experience of military helicopter aircrew," *International Journal of Industrial Ergonomics*, vol. 42, no. 1, pp. 73–79, 2012.

[95] Karuppasamy K. and Obuchowski N., "Comparison of Fit for Sealed and Loose-Fitting Surgical Masks and N95 Filtering Facepiece Respirators," *Annals of work exposures and health*, vol. 65, no. 4, pp. 463–474, 2021. doi: 10.1093/annweh/wxaa125 33458738 PMC7929389

[96] Schreinemakers J. R. C., Oudenhuijzen A. J., van Amerongen P. C., and Kon M., "Oxygen mask fit analysis in F-16 fighter pilots using 3D imaging," *Aviation*, *space*, *and environmental medicine*, vol. 84, no. 10, pp. 1029–1033, 2013. doi: 10.3357/asem.3611.2013 24261054

[97] Abdullah Z. et al., "Design and development of a flexible wearable sit-stand passive exoskeleton using quality function deployment," *Proceedings of Mechanical Engineering Research Day*, vol. 2020, pp. 308–309, 2020.

[98] Toigo S. et al., "Fit Testing Retrofitted Full-Face Snorkel Masks as a Form of Novel Personal Protective Equipment During the COVID-19 Pandemic," *Disaster Medicine and Public Health Preparedness*, pp. 1–16, 2021. doi: 10.1017/dmp.2021.133 33926606 PMC8209433

[99] Sandaradura I. et al., "A close shave? Performance of P2/N95 respirators in healthcare workers with facial hair: results of the BEARDS (BEnchmarking Adequate Respiratory DefenceS) study," *Journal of Hospital Infection*, vol. 104, no. 4, pp. 529–533, 2020. doi: 10.1016/j.jhin.2020.01.006 31978416

[100] Ling W., Houston V., Tsai Y.-S., Chui K., and Kirk J., "Women’s load carriage performance using modular lightweight load-carrying equipment," *Military medicine*, vol. 169, no. 11, pp. 914–919, 2004. doi: 10.7205/milmed.169.11.914 15605942

[101] W. H. Harper, J. J. Knapik, and R. de Pontbriand, "Equipment compatibility and performance of men and women during heavy load carriage," in *Proceedings of the Human Factors and Ergonomics Society Annual Meeting*, 1997, vol. 41, no. 1: SAGE Publications Sage CA: Los Angeles, CA, pp. 604–608.

[102] Furnell A., Molloy R., Jaffrey M., and Daniell N., "Determining the maximum acceptable length of a hard ballistic plate," *Journal of Science and Medicine in Sport*, vol. 20, p. S137, 2017.

[103] Taylor N. A., Lewis M. C., Notley S. R., and Peoples G. E., "A fractionation of the physiological burden of the personal protective equipment worn by firefighters," *European journal of applied physiology*, vol. 112, no. 8, pp. 2913–2921, 2012. doi: 10.1007/s00421-011-2267-7 22143844

[104] Coltman C. E., Brisbine B. R., Molloy R. H., and Steele J. R., "Effect of torso and breast characteristics on the perceived fit of body armour systems among female soldiers: Implications for body armour sizing and design," *Frontiers in Sport and Active Living [under review]*, 2021. 10.3389/fspor.2022.821210 PMC8959632 35356093

[105] Hsiao H., Whitestone J., Kau T.-Y., and Hildreth B., "Firefighter hand anthropometry and structural glove sizing: a new perspective," *Human factors*, vol. 57, no. 8, pp. 1359–1377, 2015. doi: 10.1177/0018720815594933 26169309 PMC4681492

[106] Kahn S. A., Leonard C., Lee Y. G., Boatwright R., Flamm T., and Woods J., "A pilot survey of Southeastern firefighters: safety practices, use of protective gear, and injury," *Burns*, vol. 46, no. 2, pp. 298–302, 2020. doi: 10.1016/j.burns.2019.03.012 31780278

[107] Brisbine B. R., Steele J. R., Phillips E. J., and McGhee D. E., "Use and perception of breast protective equipment by female contact football players," *Journal of science and medicine in sport*, vol. 23, no. 9, pp. 820–825, 2020. doi: 10.1016/j.jsams.2020.02.004 32522401

[108] McGhee D. E. and Steele J. R., "Optimising breast support in female patients through correct bra fit. A cross-sectional study," *Journal of Science and Medicine in Sport*, vol. 13, no. 6, pp. 568–572, 2010. doi: 10.1016/j.jsams.2010.03.003 20451452

[109] Coltman C. E., Steele J. R., and McGhee D. E., "Which bra components contribute to incorrect bra fit in women across a range of breast sizes?," *Clothing and Textiles Research Journal*, vol. 36, no. 2, pp. 78–90, 2018.

[110] L. L. Bossi, M. L. Jones, A. Kelly, and D. W. Tack, "A Preliminary investigation of the effect of protective clothing weight, bulk and stiffness on combat mobility course performance," in *Proceedings of the Human Factors and Ergonomics Society Annual Meeting*, 2016, vol. 60, no. 1: SAGE Publications Sage CA: Los Angeles, CA, pp. 702–706.

[111] L. Jones, G. Jenkins, M. B. Ducharme, and L. M. Bossi, "Relative contribution of bulk, stiffness, & load weight of PPE on soldier performance," in *3rd International Congress of Soldier Physical Performance. Boston, MA*, 2014.

[112] Mitchell K. B., Batty J. M., Coyne M. E., DeSimone L. L., and Bensel C. K., "Reliability analysis of time to complete the obstacle course portion of the load effects assessment program (LEAP)," ARMY NATICK SOLDIER RESEARCH DEVELOPMENT AND ENGINEERING CENTER MA NATICK …, 2016.

[113] Coltman C. E., Brisbine B. R., Molloy R. H., Ball N. B., Spratford W. A., and Steele J. R., "Identifying problems that female soldiers experience with current-issue body armour," *Applied Ergonomics*, vol. 94, p. 103384, 2021. doi: 10.1016/j.apergo.2021.103384 33690018

[114] Summers S. J., Keegan R. J., Flood A., Martin K., McKune A., and Rattray B., "The Acute Readiness Monitoring Scale: Assessing Predictive and Concurrent Validation," *Frontiers in Psychology*, p. 4223, 2021. doi: 10.3389/fpsyg.2021.738519 34630249 PMC8498198

[115] Martin K., Périard J., Rattray B., and Pyne D. B., "Physiological factors which influence cognitive performance in military personnel," *Human factors*, vol. 62, no. 1, pp. 93–123, 2020. doi: 10.1177/0018720819841757 31009241

[116] Basner M. and Dinges D. F., "Maximizing sensitivity of the psychomotor vigilance test (PVT) to sleep loss," *Sleep*, vol. 34, no. 5, pp. 581–591, 2011. doi: 10.1093/sleep/34.5.581 21532951 PMC3079937

[117] Beckner M. E. et al., "Impact of simulated military operational stress on executive function relative to trait resilience, aerobic fitness, and neuroendocrine biomarkers," *Physiology & Behavior*, vol. 236, p. 113413, 2021. 33811909 10.1016/j.physbeh.2021.113413

[118] De Bruin E. J., van Run C., Staaks J., and Meijer A. M., "Effects of sleep manipulation on cognitive functioning of adolescents: A systematic review," *Sleep medicine reviews*, vol. 32, pp. 45–57, 2017. doi: 10.1016/j.smrv.2016.02.006 27039223

[119] May B., Tomporowski P. D., and Ferrara M., "Effects of backpack load on balance and decisional processes," *Military medicine*, vol. 174, no. 12, pp. 1308–1312, 2009. doi: 10.7205/milmed-d-00-0809 20055073

[120] Scheibe S. and Blanchard-Fields F., "Effects of regulating emotions on cognitive performance: what is costly for young adults is not so costly for older adults," *Psychology and aging*, vol. 24, no. 1, p. 217, 2009. doi: 10.1037/a0013807 19290754 PMC2658623

[121] Fong D.-Y., Chi L.-K., Li F., and Chang Y.-K., "The benefits of endurance exercise and Tai Chi Chuan for the task-switching aspect of executive function in older adults: an ERP study," *Frontiers in Aging Neuroscience*, vol. 6, p. 295, 2014. doi: 10.3389/fnagi.2014.00295 25389403 PMC4211410

[122] Carter L., Russell P. N., and Helton W. S., "Target predictability, sustained attention, and response inhibition," *Brain and Cognition*, vol. 82, no. 1, pp. 35–42, 2013. doi: 10.1016/j.bandc.2013.02.002 23501702

[123] Gijsbertse K., Linssen L., Woering A., and Catoire M., "The effects of mass, bulk and stiffness of personal protective equipment and clothing on physical performance when performing a military mobility obstacle course," *Applied Ergonomics*, vol. 95, p. 103448, 2021. doi: 10.1016/j.apergo.2021.103448 33930708

[124] Fullagar H. H., Sampson J. A., Mott B. J., Burdon C. A., Taylor N. A., and Groeller H., "Employment standards for Australian urban firefighters: Part 4: Physical aptitude tests and standards," *Journal of occupational and environmental medicine*, vol. 57, no. 10, pp. 1092–1097, 2015. doi: 10.1097/JOM.0000000000000528 26461864

[125] Cheung S. S., Lee J. K., and Oksa J., "Thermal stress, human performance, and physical employment standards," *Applied physiology*, *nutrition*, *and metabolism*, vol. 41, no. 6, pp. S148–S164, 2016. 10.1139/apnm-2015-0518 27277564

[126] Fischer S. L., Sinden K. E., and MacPhee R. S., "Identifying the critical physical demanding tasks of paramedic work: Towards the development of a physical employment standard," *Applied ergonomics*, vol. 65, pp. 233–239, 2017. doi: 10.1016/j.apergo.2017.06.021 28802444

[127] Stevenson R. D., Siddall A. G., Turner P. F., and Bilzon J. L., "Implementation of Physical Employment Standards for Physically Demanding Occupations," *Journal of Occupational and Environmental Medicine*, vol. 62, no. 8, pp. 647–653, 2020. doi: 10.1097/JOM.0000000000001921 32472847

[128] Kobus D. A., Brown C. M., Wu L., Robusto K., and Bartlett J., "Cognitive performance and physiological changes under heavy load carriage," PACIFIC SCIENCE AND ENGINEERING GROUP INC SAN DIEGO CA, 2010.

[129] Eddy M. D. et al., "The effects of load carriage and physical fatigue on cognitive performance," *PloS one*, vol. 10, no. 7, p. e0130817, 2015. doi: 10.1371/journal.pone.0130817 26154515 PMC4496096

[130] Epling S. L., Blakely M. J., Russell P. N., and Helton W. S., "Free recall and outdoor running: cognitive and physical demand interference," *Experimental brain research*, vol. 234, no. 10, pp. 2979–2987, 2016. doi: 10.1007/s00221-016-4700-y 27299913

[131] Mahoney C. R., Hirsch E., Hasselquist L., Lesher L. L., and Lieberman H. R., "The effects of movement and physical exertion on soldier vigilance," *Aviation*, *space*, *and environmental medicine*, vol. 78, no. 5, pp. B51–B57, 2007. 17547304

[132] AlGhamri A. A., Murray S. L., and Samaranayake V., "The effects of wearing respirators on human fine motor, visual, and cognitive performance," *Ergonomics*, vol. 56, no. 5, pp. 791–802, 2013. doi: 10.1080/00140139.2013.767383 23514088

[133] Hancock P., "Specifying and mitigating thermal stress effects on cognition during personal protective equipment use," *Human factors*, vol. 62, no. 5, pp. 697–703, 2020. doi: 10.1177/0018720820933794 32525427

[134] Davey S. L., Lee B. J., Robbins T., Randeva H., and Thake C. D., "Heat stress and PPE during COVID-19: impact on healthcare workers’ performance, safety and well-being in NHS settings," *Journal of Hospital Infection*, vol. 108, pp. 185–188, 2021. doi: 10.1016/j.jhin.2020.11.027 33301841 PMC7720696

[135] Mahood Q., Van Eerd D., and Irvin E., "Searching for grey literature for systematic reviews: challenges and benefits," *Research synthesis methods*, vol. 5, no. 3, pp. 221–234, 2014. doi: 10.1002/jrsm.1106 26052848

[136] Paez A., "Gray literature: An important resource in systematic reviews," *Journal of Evidence‐Based Medicine*, vol. 10, no. 3, pp. 233–240, 2017. doi: 10.1111/jebm.12266 28857505

